# A gene signature is critical for intrahepatic cholangiocarcinoma stem cell self-renewal and chemotherapeutic response

**DOI:** 10.1186/s13287-022-02988-9

**Published:** 2022-07-15

**Authors:** Lifeng Huang, Dongwei Xu, Yawei Qian, Xiaoqiang Zhang, Han Guo, Meng Sha, Rui Hu, Xiaoni Kong, Qiang Xia, Yi Zhang

**Affiliations:** 1 Department of General Surgery, The First Affiliated Hospital, Nanjing Medical University, 300 Guangzhou Road, Nanjing, 210000 China; 2 Department of Liver Surgery, School of Medicine, Renji Hospital, Shanghai Jiao Tong University, 160 Pujian Road, Shanghai, 200127 China; 3 Central Laboratory, Department of Liver Diseases, ShuGuang Hospital Affiliated to Shanghai University of Chinese Traditional Medicine, 528 Zhangheng Road, Shanghai, 201203 China

**Keywords:** Cancer stem cells, Intrahepatic cholangiocarcinoma, Methionine cycle, Adjuvant chemotherapeutics, Prognosis

## Abstract

**Background:**

Improved understanding of the stemness regulation mechanism in intrahepatic cholangiocarcinoma (ICC) could identify targets and guidance for adjuvant transarterial chemoembolization (TACE).

**Methods:**

TCGA database was excavated to identify the ICC stemness-associated genes. The pro-stemness effect of target genes was further analyzed by sphere formation assay, qRT-PCR, western blot, flow cytometric analysis, IHC, CCK8 assay and metabolomic analysis. Based on multivariate analysis, a nomogram for ICC patients with adjuvant TACE was established and our result was further confirmed by a validation cohort. Finally, the effect of dietary methionine intervention on chemotherapy was estimated by in vivo experiment and clinical data.

**Results:**

In this study, we identified four ICC stemness-associated genes (SDHAF2, MRPS34, MRPL11, and COX8A) that are significantly upregulated in ICC tissues and negatively associated with clinical outcome. Functional studies indicated that these 4-key-genes are associated with self-renewal ability of ICC and transgenic expression of these 4-key-genes could enhance chemoresistance of cholangiocarcinoma cells. Mechanistically, the 4-key-genes-mediated pro-stemness requires the activation of methionine cycle, and their promotion on ICC stemness characteristic is dependent on MAT2A. Importantly, we established a novel nomogram to evaluate the effectiveness of TACE for ICC patients. Further dietary methionine intervene studies indicated that patients with adjuvant TACE might benefit from dietary methionine restriction if they have a relatively high nomogram score (≥ 135).

**Conclusions:**

Our results show that four ICC stemness-associated genes could serve as novel biomarkers in predicting ICC patient’s response to adjuvant TACE and their pro-stemness ability may be attributed to the activation of the methionine cycle.

**Supplementary Information:**

The online version contains supplementary material available at 10.1186/s13287-022-02988-9.

## Background

Intrahepatic cholangiocarcinoma (ICC), which originates from the epithelial cells lining the intrahepatic biliary tree, is a molecularly heterogeneous malignancy with limited therapeutic options [1]. Although ranking as the second most common primary hepatobiliary neoplasm inferior only to hepatocellular carcinoma, ICC remains a relatively rare cancer, with limited literature reporting only small amount of patients [2, 3]. Currently, liver resection is still the mainstay of radical treatment for ICC patients, while the prognosis after surgery is disappointing, with a 5-year survival rate of 20% to 35% [4]. Previous studies reported that adjuvant transarterial chemoembolization (TACE) might be a practical choice for selected ICC patients following hepatectomy [5–7]. However, to the best of our knowledge, there is still no reliable biomarker for ICC patients with adjuvant TACE, let alone prognosis models to evaluate the effectiveness of this very therapy. Therefore, establishing a novel predictive model for ICC patients with adjuvant TACE is urgently needed.

Accumulating evidence demonstrated that chemotherapeutic resistance is a result of cancer stem cells (CSCs), a subpopulation of cells in a tumor possess the capacity for self-renewal and generation of heterogeneous lineages [8, 9]. Many of the current chemotherapeutic strategies could eliminate most tumor cells while leaving CSCs behind, and the residual CSCs are capable of regenerating the neoplasm [10]. Therefore, from a clinical perspective, a detailed understanding of the mechanisms of chemoresistance of CSCs is expected to guide adjuvant chemotherapy for ICC patients. However, the molecular mechanisms regulating the chemoresistance of CSCs in human ICC remain elusive.

Progress on cancer-specific metabolism has opened up a new territory for clinical cancer therapy. It has been documented that CSCs, which functionally differ from non-CSCs, may exhibit distinct metabolic requirements [11, 12]. Methionine, a nutritionally indispensable amino acid, is involved in a variety of cellular functions including epigenetic modifications, redox homeostasis and tumorigenesis [13]. Recent studies suggested that methionine cycle plays an important role in maintaining CSCs features and drug resistance [14, 15]. Therefore, it is conceivable that the methionine metabolism could regulate the stemness of ICC and eventually determines the effectiveness of adjuvant chemotherapy.

In this study, we identified that four CSCs-associated genes are upregulated in ICC tissues and negatively correlated with prognosis: succinate dehydrogenase complex assembly factor 2 (SDHAF2), which encodes a mitochondrial protein required for the flavination of a succinate dehydrogenase complex subunit needed for activity of the complex [16]; mitochondrial ribosomal protein S34 (MRPS34), which encodes a 28S subunit protein and is one of 15 mammalian mitochondria-specific proteins [17]; mitochondrial ribosomal protein L11 (MRPL11), which encodes a 39S subunit component of the mitochondrial ribosome [18]; and cytochrome c oxidase subunit 8A (COX8A), which encodes the smallest subunit of cytochrome c oxidase, the terminal enzyme of the respiratory chain [19]. Functional studies further indicated that these 4-key-genes could enhance the self-renewal and chemoresistance ability of cholangiocarcinoma. In addition, multivariate analysis showed that SDHAF2, MRPL11, COX8A, serum carbohydrate antigen 19-9 (CA19-9), tumor size and lymph node metastasis are independent factors for overall survival (OS) of ICC patients with adjuvant TACE. Based on that, we established a novel nomogram (C-index, concordance index: 0.85, 0.81 to 0.89) to evaluate the effectiveness of adjuvant TACE for ICC patients. Mechanistic studies suggested that the 4-key-genes-mediated pro-stemness requires the activation of methionine cycle. Further, in vivo experiment and clinical data analysis indicated that dietary methionine restriction may improve the prognosis of certain ICC patients with adjuvant TACE (nomogram score ≥ 135), providing new insights into cancer-specific metabolic vulnerabilities in ICC.

## Methods

### Data collection and processing

The RNA-seq transcriptome data of 36 primary ICC samples and 9 non-tumor samples (33 ICC samples and 8 non-tumor samples have complete clinical information) were obtained from the TCGA database (http://cancergenome.nih.gov/). These data were current as of February 17, 2021. Next, the Ensembl database (http://asia.ensembl.org/index.html) was used to convert gene names from Ensembl IDs to a matrix of gene symbols. In addition, differentially expressed genes (DEGs) were obtained by using R package “edgeR.” The selection criteria were as follows: false discovery rate (FDR) < 0.05 and |log_2_ fold change|> 1.0. The level of mRNAsi (mRNA expression-based stemness index) and EREG-mRNAsi (epigenetically regulated mRNAsi) were detected in normal and tumor samples. The corrected mRNAsi (mRNAsi/tumor purity) and corrected EREG-mRNAsi (EREG-mRNAsi/tumor purity) were measured in tumor samples. The prognostic value of corrected mRNAsi or corrected EREG-mRNAsi was estimated by Kaplan–Meier analysis and log-rank test.

### Confirmation of significant modules and key genes

Weighted gene co-expression network analysis (WGCNA) was performed using the WGCNA R package [20, 21]. EREG-mRNAsi and mRNAsi were chosen as the sample traits to locate CSCs-related modules and genes. Modules with the highest module significance were considered as the most sample trait-related modules. After detecting modules of interest, we calculated gene significance (GS) and module membership (MM) for each gene. The thresholds for screening target genes in a certain module were defined as cor.MM > 0.8 and cor.GS > 0.5.

### Cell lines and cell culture

The cholangiocarcinoma cell line RBE and HCCC9810 were purchased from the Cell Bank of the Chinese Academy of Sciences (Shanghai, China). The primary ICC cells were gathered from fresh clinical tissue specimens. The culture medium for primary ICC cells and cholangiocarcinoma cell lines was RPMI1640 (contained 20% fetal bovine serum, 100 μg/ml penicillin G and 100 U/ml streptomycin) in a humidified cell incubator under an atmosphere of 5% CO_2_ at 37 °C.

### qRT-PCR and western blot

A total of 40 pairs of ICC tumor and paratumor collected at Renji Hospital from 2011 to 2013 were used for qRT-PCR analysis and 4 pairs of ICC specimen were collected for western blot analysis. Total RNA was isolated by TRIzol reagent (Invitrogen) and reverse-transcribed to cDNA with a cDNA Synthesis Kit (Thermo Scientific). qRT-PCR was performed on the CFX96 Real-Time PCR system with SYBR Green PCR Master Mix (Applied Bio-Rad). Primers sequences are listed in Additional file 2: Table S1. Protein extracts of ICC tissues and cholangiocarcinoma cells were analyzed by western blot according to the standard protocol provided by manufacturer.

### Target gene overexpression and shRNA-mediated interference

For stable cell line construction, lentivirus and shRNAs productions were completed by Hanbio Biotechnology Co., Ltd. (Shanghai, China) and used according to the manufacturer’s protocols. Briefly, cells were transfected with concentrated virus at a multiplicity of infection of 20 with polybrene for 6 h and then 1:1 fresh medium was added for the following 18 h. Expression of target genes in the infected cells was validated by qRT-PCR and western blot.

### Flow cytometric analysis

Cholangiocarcinoma cells were stained with PE-conjugated antihuman CD133 and APC-conjugated antihuman CD44 antibodies (eBioscience). The isotype-matched IgG was served as a control (eBioscience). The fluorescence intensity was measured on a flow cytometer (BD Biosciences). For cell sorting, PE-conjugated antihuman CD133 antibodies and APC-conjugated antihuman CD44 antibodies were incubated with cholangiocarcinoma cells, followed by sorting with a flow cytometer (BD Biosciences). In our study, CD133^+^CD44^+^ subgroup was defined as CSC, while the rest cells were defined as non-CSC.

### Spheroid formation assay

A total of 1000 single cholangiocarcinoma cell lines were plated onto 12-well ultra-low attachment culture dishes (Corning) in serum-free DMEM/F12 medium for 7 days. DMEM/F12 was supplemented with 20 ng/ml epidermal growth factor (EGF, Invitrogen), 10 ng/ml basic fibroblast growth factor (bFGF, Invitrogen), B27 (1:50; Invitrogen), N2 (1:100; Invitrogen), 1% sodium pyruvate, 100 μg/ml penicillin G and 100 U/ml streptomycin. The primary spheres were derived from fresh clinical tissue specimen and subsequently dissociated with trypsin and resuspended in DMEM/F12 medium with the above supplements. Tumorsphere-derived adherent cells were maintained in the same medium without EGF, bFGF, B27, N2 and were instead supplemented with 10% fetal bovine serum. The number of spheroids formed (≥ 50 μm) was counted under an inverted microscope (Leica).

### Cell invasion assay

The transwell chamber was coated with 1: 8 diluted Matrigel (100 μl, Corning, USA) and incubated for 5 h. Then, a total of 1 × 10^5^ cells were suspended in serum-free RPMI1640, seeded into the upper chamber and allowed to migrate toward RPMI1640 (with 10% FBS) in the lower side of the chamber for 48 h. The migrated cells were fixed in formaldehyde and stained with crystal violet. The invaded cells were counted from three random fields of microscope.

### Colony formation assay

In total, 1000 cells were seeded in 6-well plates for 14 days. After different treatment, the colonies were fixed with 10% PBS-buffered formaldehyde and stained with crystal violet to visualize the colonies.

### Chemoresistance assay

Cell viability was determined by the Cell Counting Kit-8 (CCK8) assay (DOJINDO). CCK8 assay was performed according to manufacturer’s protocols. Briefly, cells were cultured in 96-well plates at 1 × 10^4^ cells/well to attach overnight, followed by additional treatment with 5-fluorouracil (5-FU; Sigma) or cisplatin (CDDP; Sigma) at different concentrations. All experiments were based on at least three parallel measurements and each measurement contained triplicates.

### TACE therapy after surgery

ICC tissues and paired paratumor tissues of primary cohort were obtained from 71 ICC patients treated with adjuvant TACE (1–2 months after surgery) at Renji Hospital (Shanghai, China); the validation cohort contained 34 cases gathered from the same institution. The detailed clinical pathological characteristics of the patients are presented in Additional file 2: Table S2. The regimen for the adjuvant TACE consisted of 5-FU 0.75 g, mitomycin C 16 mg, CDDP 60 mg and lipiodol 5 ml. In our study, all clinical samples and patient information were obtained following informed consent and protocols which were approved by the ethical review committee of the WHO Collaborating Center for Research in Human Production (authorized by Shanghai Municipal Government).

### Tissue microarray (TMA) and immunohistochemistry (IHC)

A total of 71 samples of ICC specimens (primary cohort) collected at Renji Hospital from 2011 to 2015 were used to construct the TMA slice I. Another 34 ICC tissue samples (validation cohort) gathered in Renji Hospital were used to build the TMA slice II (2015–2017). Then, the TMA slices were subject to immunohistochemical staining according to standard protocols using specific antibodies against MRPL11 (Cell Signaling Technology); SDHAF2, MRPS34 and COX8A (Invitrogen). The staining score was assessed by the percentage of positively stained area and staining intensity. Two experienced pathologists calculated the IHC score independently, and the final IHC score was averaged from both pathologists.

### Metabolomic analysis and sample preparation

For suspension samples, cells were collected and quenched with four volumes of sodium chloride solution (150 mM), vortex mixing 1 min, 3000 g centrifugation for 5 min at 4 °C, and then discarded the supernatant. For adherent samples, after discarding the culture medium, the surface of the plates was washed by sodium chloride solution (150 mM) and then released adherent cells from the plate surface by a cell scraper. Cell pellets were collected as described before. Polar metabolites (aqueous fraction) and lipid species (organic fraction) in cell pellets were separated by a two-phase liquid–liquid extraction as described previously [14, 22]. LC–MS analysis was conducted by using a UPLC system (Waters Corp) and a mass spectrometer (Thermo Fisher Scientific).

### Methionine cycle inhibition assay

Vector cells and lentivirus transfected cells (Lv-SDHAF2, Lv-MRPS34, Lv-MRPL11 and Lv-COX8A) were treated with FIDAS-5 (5 μM final concentration, Merck Millipore), and the mRNA level of pluripotent transcription factors was further analyzed by qRT-PCR.

### Methionine-restricted diet nude mice model

For in vivo methionine-restricted diet nude mice model, 3-week-old mice (male BALB/c nude mice) were subjected to either the control or the methionine-restricted diet. Two weeks after that, bearing tumor cells (derived from either control RBE cells or RBE cells with overexpression of 4-key-genes) were implanted subcutaneously into the left flank of nude mice. Then, the mice were intraperitoneally injected with CDDP (5 mg/kg body weight), twice a week for 3 weeks. Tumor growth was calculated by the following formula: *V* = 0.5 × *W*^2^ × *L*. All procedures involving animals were approved and performed in accordance with the Animal Care and Use Committee of Shanghai Jiaotong University.

### Methionine dietary and methionine supplementary therapy

Methionine intake was gathered by analyzing daily diet of ICC patients. Low-methionine diet was defined as less than 8.6 mg kg^−1^ day^−1^, about 50% reduction in daily methionine intake. High-methionine diet was defined as more than 8.6 mg kg^−1^ day^−1^. Methionine supplementary therapy was defined as ≥ 3 days amino acids supplement following adjuvant TACE, containing 2.25 g/day methionine supplement (intravenous drip). Informed consent was obtained from each patient, and protocols were approved by the ethical review committee mentioned before.

### Statistical analysis

The statistical analysis was carried out using SPSS 20.0 and GraphPad Prism 7. The Student’s t test or Pearson’s chi-square test was used to compare the difference of clinicopathologic features between two groups. The Kruskal–Wallis test was conducted as a nonparametric test when appropriate. Survival and tumor recurrence rates were analyzed using Kaplan–Meier analysis and log-rank test. Univariate and multivariate analyses were performed based on a Cox proportional hazard regression. A nomogram was built based on the independent factors for OS. The nomogram accuracy was evaluated by C-index and calibration curves. The cutoff point of nomogram was set as 135 (3-year overall survival = 50%). Bootstraps with 2,000 resamples were used to stabilize our results. All statistical tests were two-sided, and a p value of < 0.05 (*), < 0.01 (**), or < 0.001 (***) was considered significant.

## Results

### mRNAsi and its clinical characteristics in ICC

**Fig. 1:**
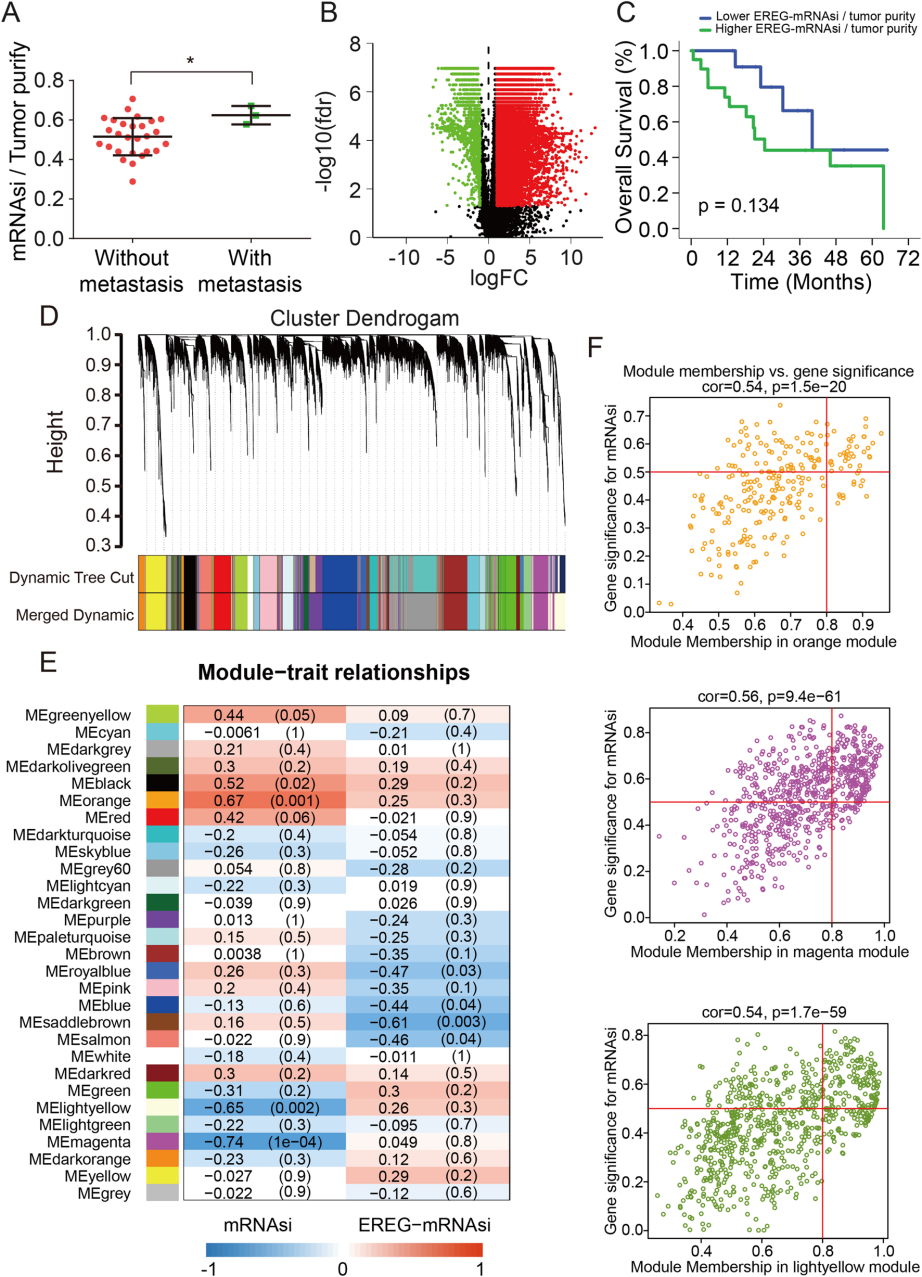
mRNAsi expression and associated key modules in human ICC. **A** Comparison of corrected mRNAsi in ICC patients with or without distant metastasis. **B** Volcano map of differentially expressed genes (DEGs). **C** Kaplan–Meier analysis of the overall survival of ICC patients in correlation with corrected EREG-mRNAsi expression in TCGA database. **D** Cluster dendrogram of genes in ICC patients; each color represents a co-expression module which contains a subgroup of highly connected genes. **E** Correlation between gene modules and mRNAsi scores (or EREG-mRNAsi scores); the correlation coefficient (left) and *p* value (right) were listed, respectively. **F** Scatter diagram for three important gene modules: the orange, magenta and light yellow module. (*p < 0.05)

To determine whether mRNAsi is associated with ICC development, we first examined mRNAsi level in ICC patients in TCGA database. Although no significant difference was found between normal and tumor tissues, mRNAsi seems to be highly expressed in ICC patients with distant metastasis (Fig. 1A, Additional file 1: Fig. S1A). Of note, mRNAsi expression was slightly higher in male than female (Additional file 1: Fig. S1B). To further evaluate differentially expressed genes in ICC tissues, DEGs screening was performed (Fig. 1B), and heatmap of the first 20 upregulated and downregulated key genes is present in Additional file 1: Fig. S1D. Probably due to the small sample size, although corrected EREG-mRNAsi is negatively associated with overall survival, no statistical significance was reached (Fig. 1C, Additional file 1: Fig. S1C). These findings suggested that mRNAsi may highly expressed in ICC patients with malignant characteristics and probably correlated with poor prognosis.

### Vital modules and genes indicated by WGCNA

**Fig. 2:**
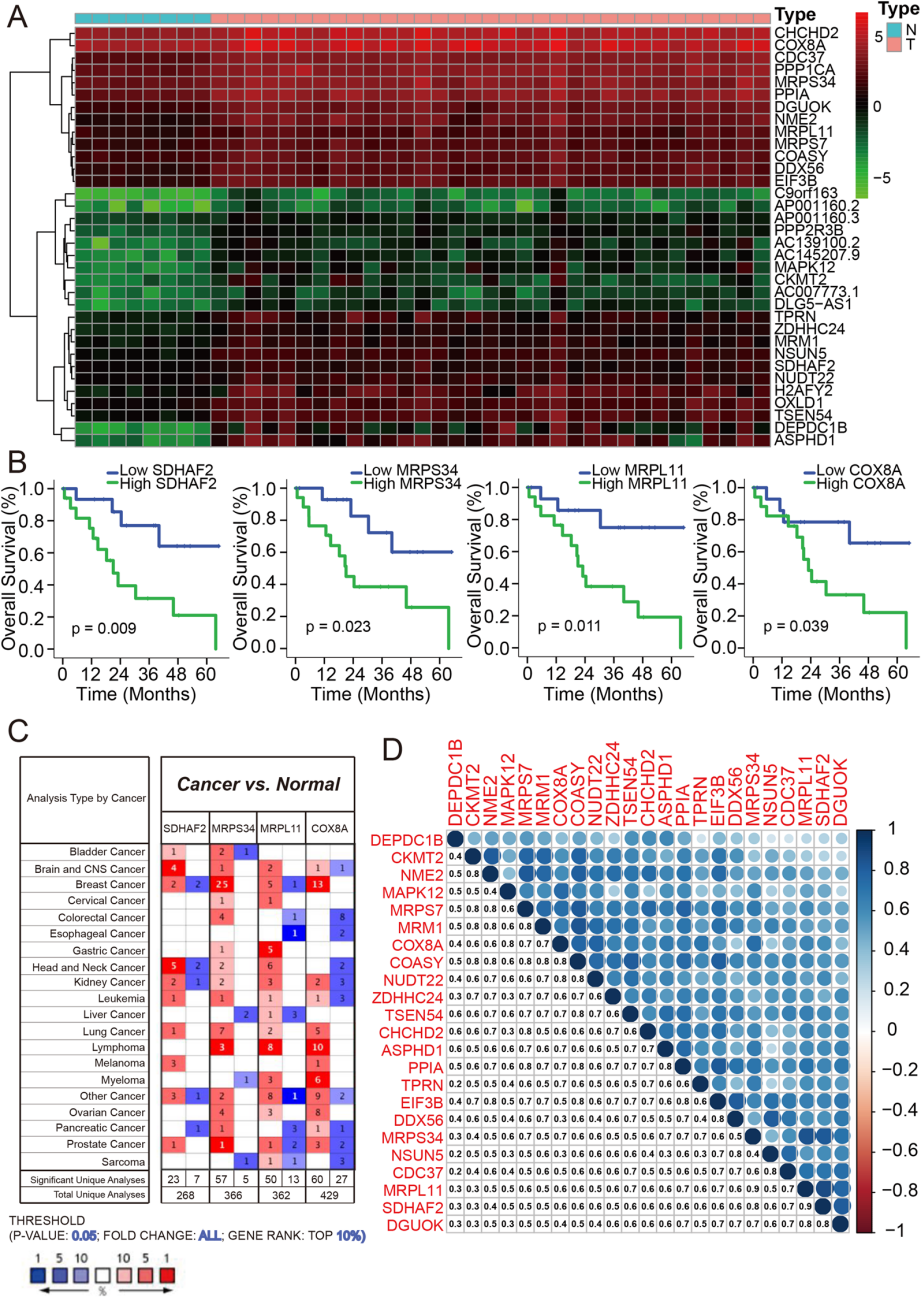
Identification of the 4-key-genes correlated with ICC prognosis. **A** Heatmap of the key genes in orange module. **B** Kaplan–Meier curve of the overall survival of ICC patients in correlation with 4-key-genes expression in TCGA database. **C** The mRNA expression of 4-key-genes in multiple cancer types from Oncomine database. The number in the colored cell represents the number of analyses meeting these thresholds. The red cells indicate that the genes are higher in tumor tissues than in normal tissues, while blue cells indicate an opposite expression pattern. **D** Correlation between key genes at the transcriptional level

WGCNA was performed to construct a DEGs co-expression network, which could identify the biologically significant gene modules and further explore genes strongly linked to ICC stemness. A soft threshold (*β* = 8, scale-free *R*^2^ = 0.90) was used to ensure a scale-free network (Additional file 1: Fig. S2) and identified 29 modules for subsequent analyses (Fig. 1D–E). As shown in Fig. 1E, the orange module exhibited the highest positive association with mRNAsi, with a correlation close to 0.7, while the magenta and light yellow modules reflected a negative correlation with mRNAsi (*R*^2^ =  − 0.74, *p* < 0.001; *R*^2^ =  − 0.65, *p* = 0.002). In this study, we focus on the module positively correlated with mRNAsi; therefore, the orange module was considered as the module of greatest interest. In our study, the threshold for screening target genes in the mRNAsi group was defined as cor.MM > 0.8 and cor. GS > 0.5 (Fig. 1F). Eventually, 34 genes correlated with orange module were defined as target genes (Fig. 2A, Additional file 1: Fig. S1E). Further assessment by the Kaplan–Meier survival curve showed that patients with high expression of 4-key-genes (SDHAF2, MRPS34, MRPL11 and COX8A) exhibit significantly poor clinical outcome (Fig. 2B). Thus, these four genes were identified as the key genes correlated with ICC prognosis and included our study for subsequent analyses. To verify that the 4-key-genes could be independent factors for ICC prognosis, we also overexpressed the four genes separately in non-CSC and found no transcriptional relation to these four genes (Additional file 1: Fig. S3). Further, Oncomine database analysis indicated that these genes were overexpressed not only in ICC but also in many other types of cancer (Fig. 2C), and the correlation among the stemness-associated genes at transcriptional level is further listed in Fig. 2D.

### Role of 4-key-genes in cholangiocarcinoma stemness

**Fig. 3:**
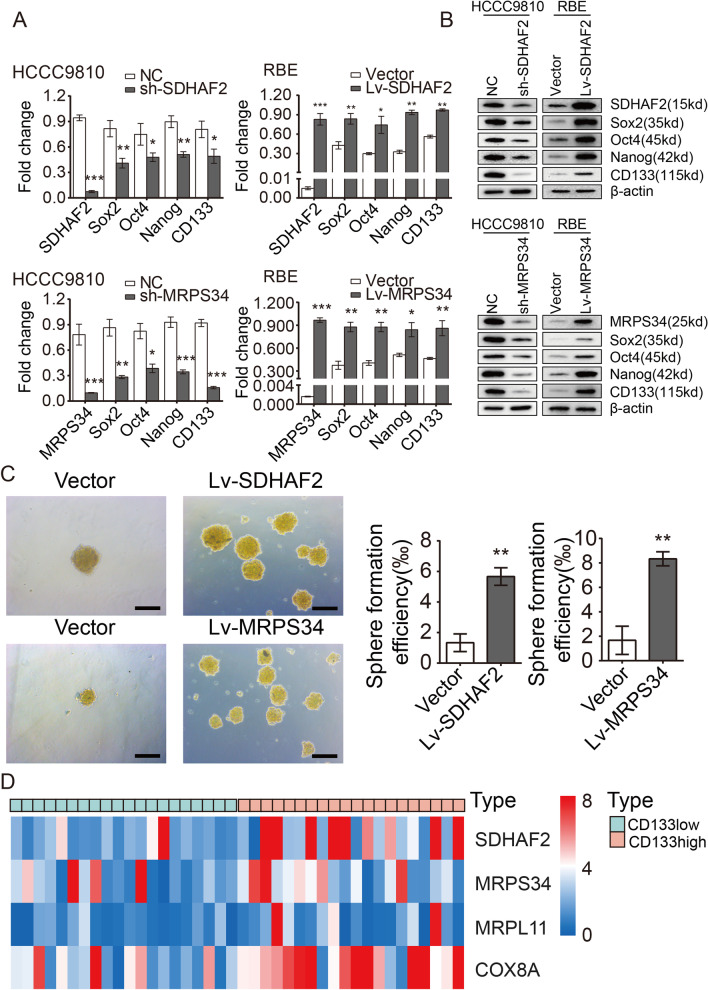
4-key-genes are associated with stemness maintenance of cholangiocarcinoma CSCs. **A** and **B** Pluripotent transcription factors were analyzed in SDHAF2 (and MRPS34) depletion or overexpression cells by qRT-PCR (**A**) and western blot (**B**). **C** SDHAF2 or MRPS34 overexpression caused an enhanced oncosphere-forming capacity in RBE cells; the right panel represents statistical results as means ± SD; Scale bar, 100 μm. **D** The transcriptional level of 4-key-genes in 40 ICC tissues. (*p < 0.05, **p < 0.01, ***p < 0.001)

To determine the role of 4-key-genes in cholangiocarcinoma CSCs, we first knocked down SDHAF2, MRPS34, MRPL11 and COX8A in HCCC9810 cell and overexpressed them in RBE cell, respectively. (The expression of stemness markers and methionine metabolites in RBE and HCCC9810 is presented in Additional file 1: Fig. S4.) Then, qRT-PCR and western blot assays were performed to analyze the expression of pluripotent transcription factors (Sox2, Oct4, Nanog and CD133). Of note, 4-key-genes depletion dramatically reduced the expression of pluripotent transcription factors, whereas 4-key-genes overexpression significantly promoted their expression in cholangiocarcinoma cells (Fig. 3A and B, Additional file 1: Fig. S5A–C). Further 4-key-gene modulation experiments in RBE and HCCC9810 are presented in Additional file 1: Fig. S6. What is more, we also discovered that higher 4-key-genes expression ensured an enhanced spheroid formation ability in cholangiocarcinoma cells (Fig. 3C and Additional file 1: Fig. S5D). Consistently, 4-key-genes enhancement significantly increased CD133^+^/CD44^+^ population in RBE cell, and CSC isolated from primary ICC cells exhibits high expression of 4-key-genes compared to non-CSC (Additional file 1: Fig. S7 and S8). Further function study indicated that 4-key-genes could also enhance Wnt pathway and promote ICC proliferation (Additional file 1: Fig. S9). In addition, correlation analysis between 4-key-genes and CD133 level in ICC clinical specimen demonstrated that 4-key-genes expression was preferentially observed in CD133 high group (Fig. 3D). These findings strongly indicated that these 4-key-genes are required for stemness maintenance of cholangiocarcinoma CSCs.

### 4-key-genes enhance the chemoresistance of cholangiocarcinoma cells

**Fig. 4:**
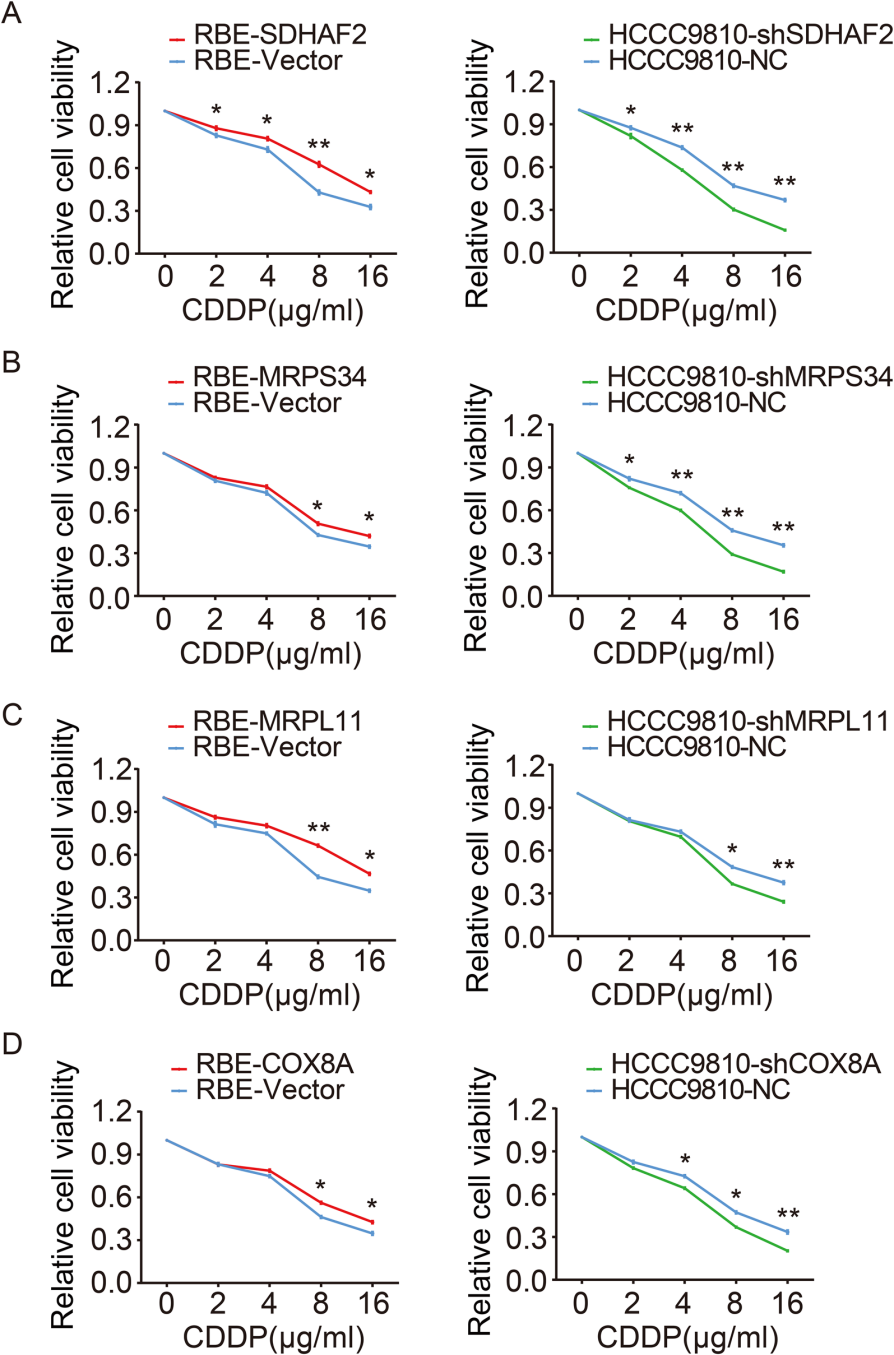
4-key-genes enhance chemoresistance of ICC. **A** Cell viability was analyzed in SDHAF2 depletion and overexpression cholangiocarcinoma cells after treatment with CDDP. **B** Cell viability was analyzed in MRPS34 depletion and overexpression cholangiocarcinoma cells after treatment with CDDP. **C** Cell viability was analyzed in MRPL11 depletion and overexpression cholangiocarcinoma cells after treatment with CDDP. **D** Cell viability was analyzed in COX8A depletion and overexpression cholangiocarcinoma cells after treatment with CDDP. Data are shown as means ± SD. (*p < 0.05, **p < 0.01)

CSCs are defined as a group of cells in a neoplasm that possess the properties of tumorigenicity and self-renewal, which are highly responsible for chemoresistance and tumor recurrence [23, 24]. Considering the importance of CSCs in chemoresistance and the role of 4-key-genes in CSCs stemness maintenance, we next investigated the significance of 4-key-genes in chemotherapy. CCK8 assays showed that 4-key-genes enhancement leads to dramatic drug resistance to CDDP in RBE cells. In contrast, depletion of 4-key-genes sensitized HCCC9810 cells to CDDP treatment (Fig. 4A–D). Consistently, similar chemoresistance enhancement can be observed in 5-FU treatment (Additional file 1: Fig. S10A–D). Taken together, 4-key-genes could enhance chemoresistance to both CDDP and 5-FU treatment of cholangiocarcinoma cells.

### Identification of independent biomarkers for ICC patients with adjuvant TACE

**Fig. 5:**
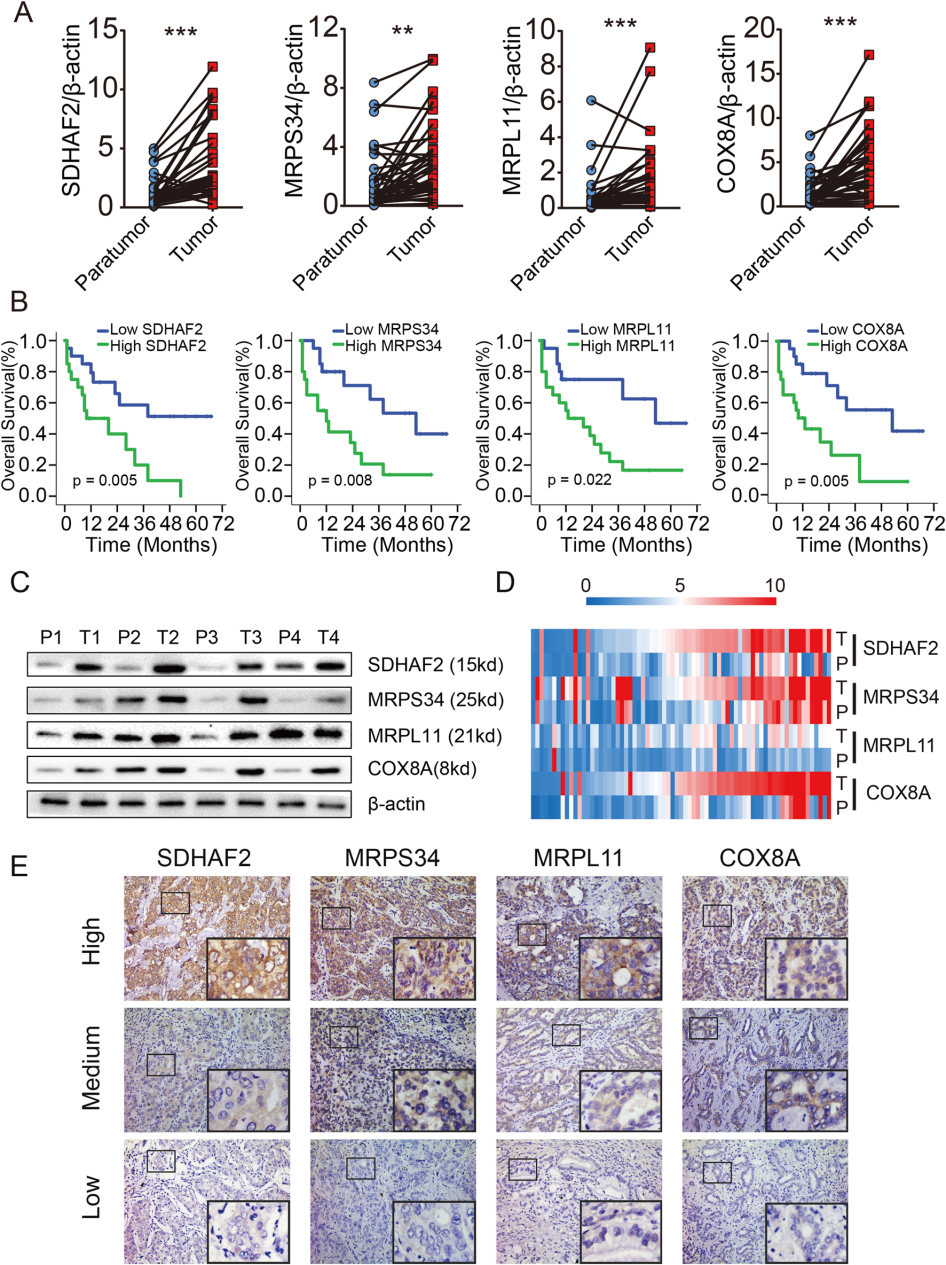
4-key-genes are poor prognostic predictors for ICC patients with adjuvant TACE. **A** The transcriptional levels of 4-key-genes were evaluated via qRT-PCR in 40 paired ICC specimens. **B** Kaplan–Meier curve suggested that ICC patients with adjuvant TACE tend to have poor overall survival if they had a high transcriptional level of the 4-key-genes. **C** Representative western blot analysis of ICC tumor tissues and paired paratumor tissues. **D** The 4-key-genes expression in TMA (71 ICC patients with adjuvant TACE). **E** Representative immunohistochemical images of 4-key-genes expression in TMA (magnification, 200 ×). (**p < 0.01, ***p < 0.001; T, tumor; P, paratumor)

**Fig. 6:**
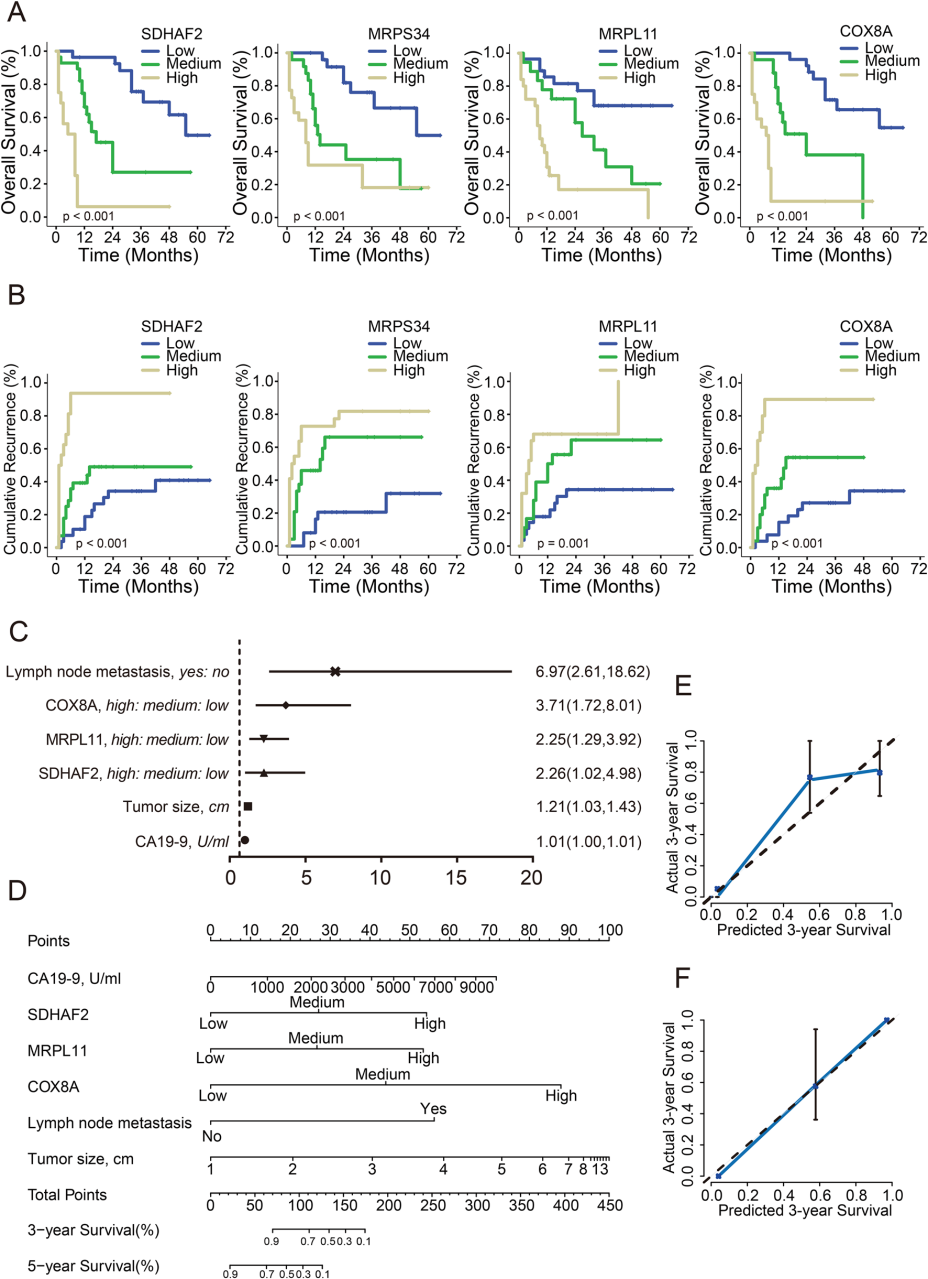
Construction and validation of nomogram for ICC patients with adjuvant TACE. **A** and **B** Kaplan–Meier analysis of the overall survival (**A**) and cumulative recurrence (**B**) in correlation with 4-key-genes expression in primary cohort. **C** Forest map of independent factors associated with OS in ICC patients with adjuvant TACE. **D** The survival nomogram of ICC patients with adjuvant TACE. **E** and **F** The calibration curve for predicting patient survival at 3 years in the primary (**E**) and validation (**F**) cohort

To further characterize the 4-key-genes in clinical ICC specimen, we explored their expression in 40 pairs of ICC tumor and adjacent paratumor tissues. As shown in Fig. 5A, assessment of qRT-PCR indicated that the 4-key-genes were highly expressed in ICC tumor tissues compared with matched paratumor tissues. Kaplan–Meier analysis on the 40 cases ICC cohort suggested that ICC patients with adjuvant TACE tend to have an unfavorable prognosis if they had a high transcriptional level of 4-key-genes (Fig. 5B, Additional file 1: Fig. S11). In addition, both western blot and immunohistochemistry (IHC) of the ICC tissue microarray (TMA) showed that the 4-key-genes were enriched in ICC tumor tissues (Fig. 5C and D). Consistently, patients with high level of 4-key-genes are correlated with poor overall survival and high risk of tumor recurrence (Fig. 5E, Fig. 6A and B). Further univariate and multivariate assessment of prognosis-related risk factors indicated that CA19-9 (*p* = 0.003), tumor size (*p* = 0.023), lymph node metastasis (*p* < 0.001), SDHAF2 expression level (*p* = 0.044), MRPL11 expression level (*p* = 0.004) and COX8A expression level (*p* = 0.001) were independent factors for OS of ICC patients with adjuvant TACE (Fig. 6C, Additional file 2: Table S3). Our findings were further confirmed by the validation cohort recruited at the same institution (*n* = 34) (Additional file 1: Fig. S12).

### Construction and validation of nomogram for ICC patients with adjuvant TACE

To our best knowledge, currently there is no prediction model available that could accurately predict the prognosis of ICC patients following adjuvant TACE. Therefore, a clinical model is urgently required to identify patients who might benefit most from this very therapy. Since multivariate analysis suggested that CA19-9, tumor size, lymph node metastasis, SDHAF2, MRPL11 and COX8A are independent factors for OS of ICC patients with adjuvant TACE, a nomogram that included all these risk factors was constructed (C-index: 0.85, 0.81–0.89) (Fig. 6D). The calibration curve for the probability of 3-year survival demonstrated a pretty good agreement between the prediction by nomogram and actual observation (Fig. 6E). The validation cohort (*n* = 34) from the same institution further guaranteed our results: The C-index of the validation cohort for predicting OS was 0.88 (95% CI, 0.82 to 0.95), and the calibration curve revealed an optimal agreement between prediction and actual observation in the probability of 3-year survival (Fig. 6F).

### 4-key-genes promote ICC stemness features in a MAT2A-dependent manner

**Fig. 7:**
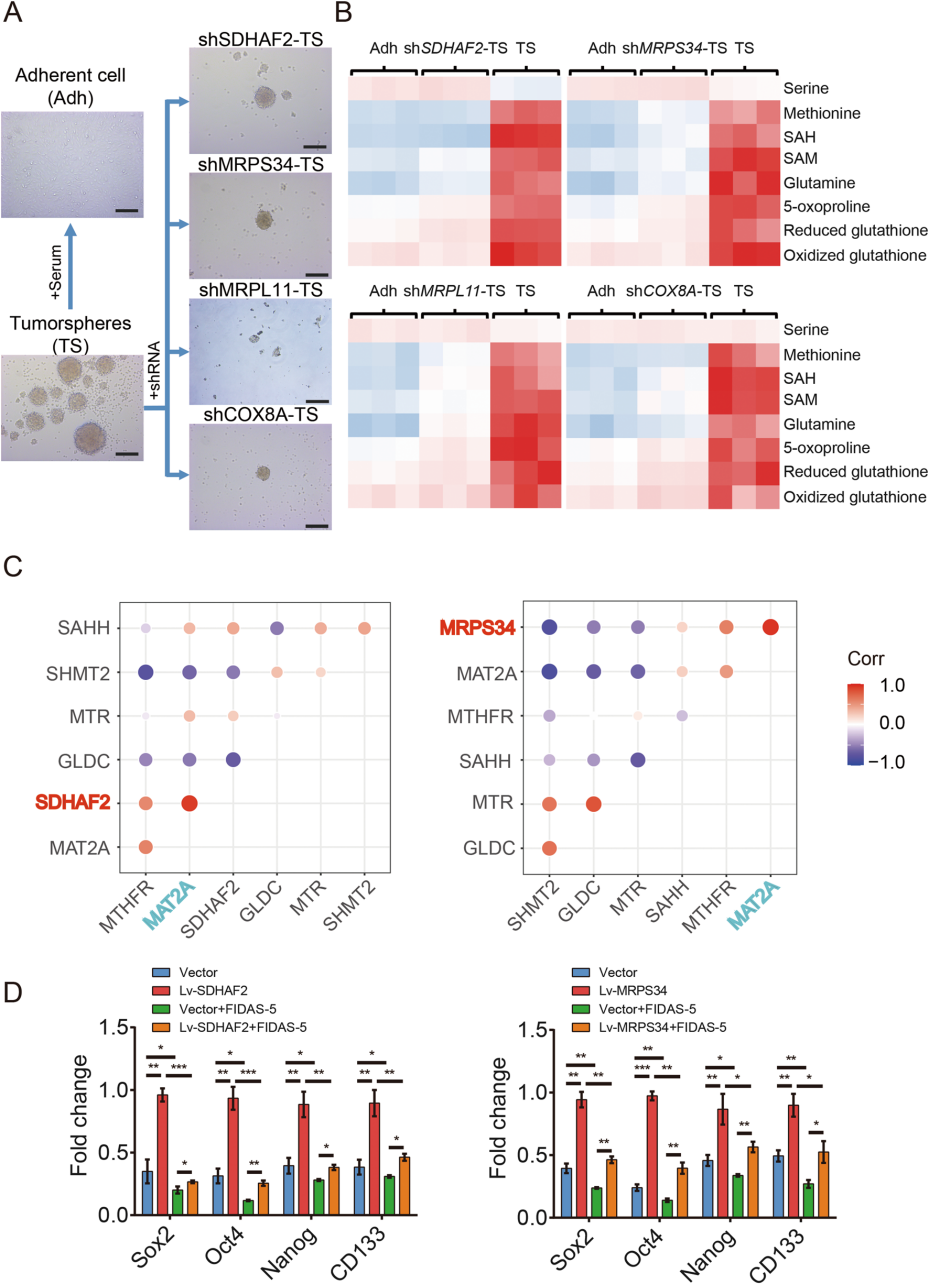
4-key-genes promote ICC stemness features in a MAT2A-dependent manner. **A** Tumorspheres (TS) of primary ICC cells were treated with serum (adherent cell, Adh) or shRNA hairpin against SDHAF2, MRPS34, MRPL11 and COX8A (shSDHAF2-TS, shMRPS34-TS, shMRPL11-TS and shCOX8A-TS). Scale bar, 100 μm. **B** Methionine metabolomic comparison in different subgroups of primary ICC cells. **C** The correlation between SDHAF2 (or MRPS34) and key enzymes of methionine cycle in primary ICC cells. **D** Vector and Lv-SDHAF2 HCCC9810 cells (or Lv-MRPS34 HCCC9810 cells) were treated with or without FIDAS-5, and then the expression of pluripotent transcription factors was analyzed by qRT-PCR. (*p < 0.05, **p < 0.01, ***p < 0.001)

Methionine is reported to be a metabolic dependency of tumor-initiating cells [14]. To further illustrate the molecular mechanism by which 4-key-genes promote stemness in cholangiocarcinoma cells, we then focused on methionine metabolism. Compared to parental primary tumorspheres, both adherent cells and 4-key-genes knockdown cells showed a strikingly decrease in oncosphere formation capacity as well as reduction in methionine cycle activity (Fig. 7A and B). Moreover, the transcriptional level of MAT2A was significantly correlated with that of 4-key-genes in primary ICC cells (Fig. 7C, Additional file 1: Fig. S13A and B). To further illustrate whether methionine cycle activation contributes to 4-key-genes-mediated stemness enhancement in cholangiocarcinoma cells, FIDAS-5 (the MAT2A inhibitor) was used in cholangiocarcinoma cells in the context of 4-key-genes overexpression. Just as we had expected, inhibition of MAT2A greatly impaired 4-key-genes-mediated enhancement of stemness in cholangiocarcinoma cells (Fig. 7D, Additional file 1: Figs. S13C, S14). Since Wnt pathway plays a pivotal role in maintaining the stemness of CSC, we further detected the Wnt pathway activity and methionine metabolites in CSC and non-CSC. We discovered that Wnt pathway is activated in CSC compared to non-CSC, and the inhibition of methionine cycle could attenuate the Wnt pathway activity both in CSC and in non-CSC cells (Additional file 1: Fig. S15). Taken together, these findings revealed that activation of methionine cycle is required for 4-key-genes-mediated stemness enhancement.

Interestingly, we then inhibit the methionine cycle in four genes overexpression ICC cells and found that the invasion ability was impaired (Additional file 1: Fig. S16A), which indicated that the methionine inhibition could attenuate the EMT enhancement by four genes. Consistently, the mRNA analysis of E-cadherin, N-cadherin and vimentin in both four genes overexpression cells and four genes overexpression cells with FIDAS-5 confirmed our results (Additional file 1: Fig. S16B).

### Dietary methionine restriction may improve the prognosis of ICC patients with high nomogram score

**Fig. 8:**
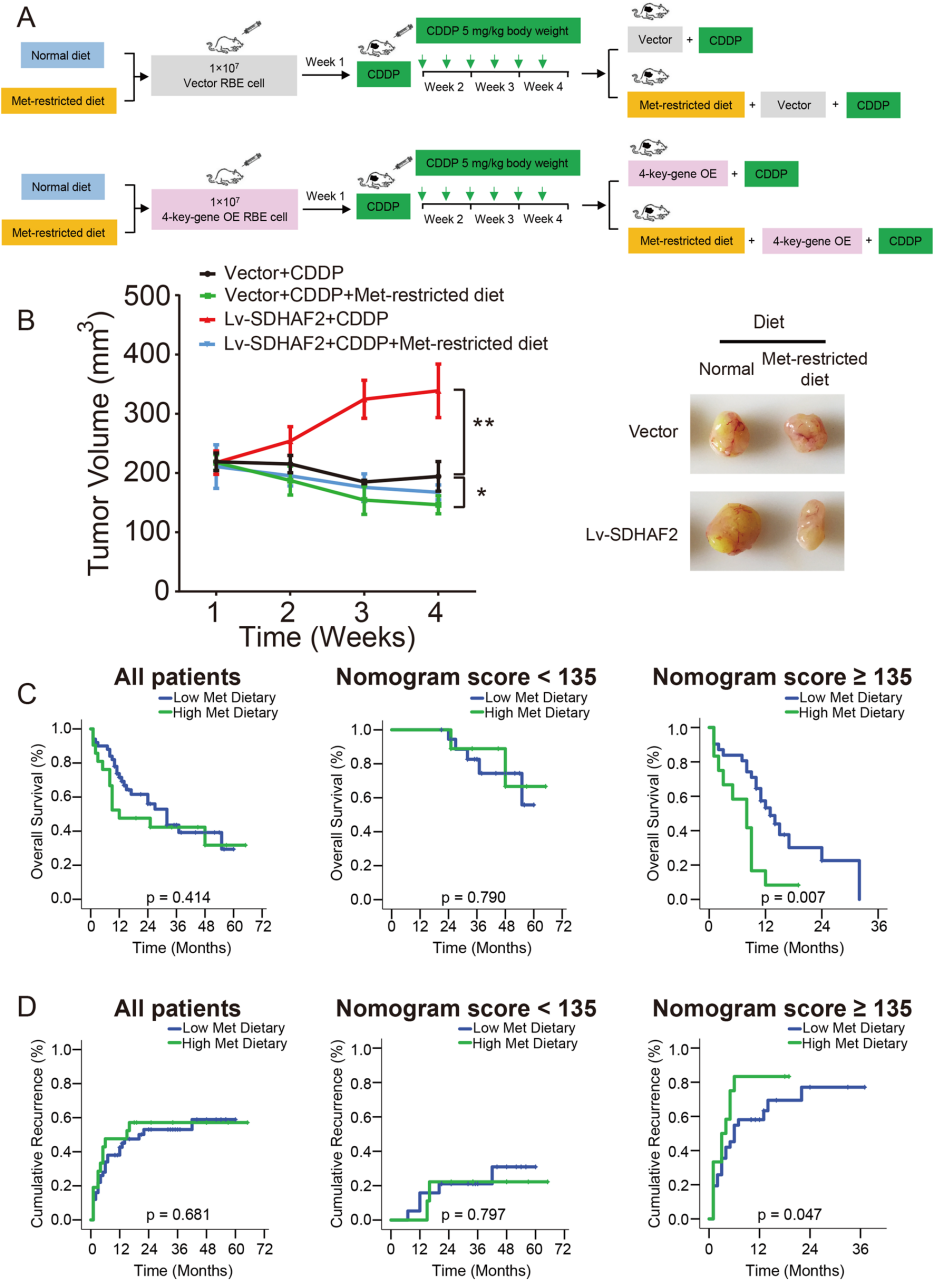
Dietary methionine restriction improves therapeutic effect of adjuvant TACE in ICC with high nomogram score. **A** Schematic representation of in vivo nude mice model. **B** The tumor growth curves of each group were summarized (Left), and the right panel shows the representative image of tumors in nude mice (Right). **C** Comparison of overall survival rates between patients with low and high-methionine dietary in primary cohort. **D** Comparison of cumulative recurrence rates between patients with low and high-methionine dietary in primary cohort. (All patients in primary cohort, *n* = 71; patients with nomogram score < 135, *n* = 28; patients with nomogram score ≥ 135, *n* = 43)

Since we found that 4-key-genes mediated the stemness of cholangiocarcinoma in a methionine metabolism-dependent way, we then started to examine whether dietary methionine restriction could influence the chemotherapeutic response of ICC. As shown in Fig. 8A and B and Additional file 1: Fig. S17, in the context of CDDP treatment, methionine-restricted diet could reduce tumor volume and growth rate of cholangiocarcinoma cells, which suggested that dietary methionine restriction could promote the chemotherapeutic response of cholangiocarcinoma cells. Interestingly, this trend becomes even more pronounced when the 4-key-genes are overexpressed. Next, we analyzed the treatment effect of methionine restriction or supplement in ICC patients with adjuvant TACE. Survival stratification was carried out by dividing patients into two groups based on nomogram score (Additional file 1: Fig. S18). The detailed clinicopathologic characteristics of these two groups are listed in Additional file 2: Table S4. The Kaplan–Meier survival analysis indicated that low-methionine dietary is associated with a good prognosis in ICC patients with nomogram score ≥ 135 (Fig. 8C and D, Additional file 1: Fig. S19). However, transient methionine therapy (≥ 3 days amino acids supplement, containing 2.25 g/day methionine, intravenous drip) fails to influence the clinical outcome of ICC patients (Additional file 1: Fig. S20). To sum up, our findings indicated that ICC patients with nomogram score ≥ 135 might benefit most from adjuvant TACE therapy if they have long-term dietary methionine restriction.

## Discussion

CSCs, in which only a small fraction of tumor cells possess the self-renewal and tumor initial capacities, are considered as the origin of chemoresistance and tumor recurrence [9, 23]. This is due to the fact that traditional anti-cancer therapies are usually insufficient to eradicate the neoplasm and the residual CSCs may prompt tumor relapse and chemoresistance [25, 26]. Growing evidence revealed that CSCs play a vital role in the development of various solid tumors, including ICC [7, 27–30]. Therefore, novel approaches targeting CSCs may be capable of overcoming these resistance mechanisms and developing effective therapies for ICC.

Cell metabolism in CSCs is a brand-new area in CSC researches with immense therapeutic potential and limited literature. Accumulating evidence suggested that cancer metabolism, particularly CSCs metabolism, could impact pathological processes and prognosis of malignancy [14, 31, 32]. Thus, novel anti-cancer strategies to systematically target on CSCs metabolism in ICC seem a potential direction in the development of cancer therapies. A recent study by Wang et al. indicated that methionine cycle activity in CSCs is distinct from non-CSCs and the inhibition of the methionine metabolism, even transiently, is sufficient to impair the self-renewal ability of CSCs [14]. What is more, Gao and his fellows demonstrated that dietary methionine restriction can produce therapeutic responses in chemoresistant models [15]. Taken together, methionine metabolism in CSCs seems to be a potential therapeutic targets. In our study, we demonstrated that CSCs in cholangiocarcinoma have different methionine metabolic features, and 4-key-genes (SDHAF2, MRPS34, MRPL11 and COX8A) could promote ICC stemness in a MAT2A-dependent manner. These results are consistent with the recent findings showing that the level of MAT2A impinges upon the sensitivity of CSCs to chemotherapy [14].

Although hepatectomy is still the first-line and most effective treatment for ICC patients, the long-term prognosis is far from satisfactory [33–35]. Previous studies suggested that adjuvant treatment in ICC patients following curative intent resection seems a practical option to improve surgical outcome [36–38]. However, reports on the adjuvant TACE effectiveness on ICC patients as well as associated biomarkers are unsatisfactory. Some recent studies demonstrated that adjuvant treatment only benefits a small group of ICC patients with certain clinical characteristics [5, 6]. Therefore, it is of importance to identify the prognostic predictors for ICC patients after adjuvant TACE and comprehensively evaluate the effectiveness of this very therapy. In our study, we discovered that 4-key-genes are associated with self-renewal ability of ICC and their overexpression could enhance chemoresistance of cholangiocarcinoma cells. Subsequent analysis further identified that these 4-key-genes could serve as reliable biomarkers for ICC patients following adjuvant TACE.

Adjuvant therapy might improve surgical outcome of ICC with R0 resection, although the hypothesis is largely based on limited retrospective analysis [39, 40]. It is believed that prediction models could potentially in favor of identification of ICC patients who might benefit most from such treatment. However, currently, the prognostic models to evaluate adjuvant TACE effectiveness on ICC patients are scarce. A recent nomogram constructed by Hyder et al. [41] only included a series of clinical–pathological variables and revealed relatively good predictive ability for ICC patients following liver resection (C-index, 0.69). Their prediction model was further evaluated by Doussot et al. [42], who demonstrated that it can be used for decision-making of adjuvant therapy. Moreover, another research by Wang et al. [34] established a nomogram (C-index, 0.74) that included clinical–pathological variables with CEA and CA19-9 to predict the prognosis of ICC after partial hepatectomy. Their recent study further indicated that this ICC nomogram can also be utilized to select candidates who might be suitable for adjuvant TACE [5]. However, the two models above only included the clinical variables with no gene signature. In addition, their prediction models, although providing some predictive power, are not specifically developed for ICC patients with adjuvant TACE. Therefore, it is conceivable that the predictive accuracy of these two systems might be hampered by these defects. To address these issues, a novel nomogram (C-index: 0.85) that is specifically aimed to evaluate the effectiveness of adjuvant TACE for ICC patients was constructed in our study.

Growing evidence has shown that methionine-restricted diet influences chemotherapeutic response in mouse tumor models and can be used to alter human cancer prognosis [15, 43, 44]. In addition, we found that 4-key-genes could improve the stemness of cholangiocarcinoma in a methionine cycle-dependent manner. Therefore, it is conceivable that the long-term dietary methionine restriction or transient methionine supplement may influence the tumor stemness and prognosis of ICC. Consistently, in vivo experiment suggested that methionine-restricted diet could improve the chemotherapeutic response of cholangiocarcinoma cells, and this trend becomes more evident when 4-key-genes are overexpressed. What is more, our adjuvant TACE data further demonstrated that methionine-restricted diet could improve the prognosis of ICC patients with high nomogram score (≥ 135), which first open up a novel therapeutic regimen for ICC with adjuvant TACE.

Our study has limitations: (1). Our prediction model was based on data gathered from a single institution in Asia, whether it can be applied to Western patients remains to be further validated; (2). In mechanistic study, we demonstrated that 4-key-genes could promote ICC stemness in a MAT2A-dependent manner, whether this CSCs specific metabolism requirements can be used in clinical application needs further investigation; (3). In dietary analysis, our assessment of methionine intake is largely based on the daily diet information gathered from patients and their relatives, which might not be precise enough. Further randomized controlled trials are needed to validate our findings.

## Conclusions

In summary, we found that four stemness-associated genes could serve as effective biomarkers for predicting ICC prognosis. Then, a novel nomogram including these biomarkers was constructed to specifically evaluate adjuvant TACE effectiveness on ICC patients. Importantly, functional experiments further indicated that their pro-stemness effect is due to methionine cycle activity enhancement. We even found that, given dietary methionine restriction, ICC patients with high nomogram score (≥ 135) might benefit most from adjuvant TACE, which provides novel perspective into metabolic intervene in ICC therapy.

## Supplementary Information

**Additional file 1** Figure S1: mRNAsi expression and associated key genes in ICC. Figure S2: Different soft threshold was analyzed to ensure a scale-free network. Figure S3: The correlation of 4-key-genes in non-CSC. Figure S4: The stemness and methionine cycle activity in ICC cell line. Figure S5: MRPL11 and COX8A are associated with stemness maintenance of cholangiocarcinoma CSCs. Figure S6: Pluripotent transcription factors were analyzed in various conditions. Figure S7: Flow cytometry analysis showed the overexpression of 4-key-genes could increase CD133+/CD44+ population in RBE cell. Figure S8: 4-key-genes level in CSC and non-CSC. Figure S9: The involvement of 4-key-genes in tumor proliferation. Figure S10: Cell viability was analyzed in 4-key-genes depletion and overexpression after treatment with 5-Fu. Figure S11: ICC with adjuvant TACE tends to recurrence when having high transcriptional level of the 4-key-genes. Figure S12: Kaplan–Meier analysis of the overall survival and cumulative recurrence in correlation with 4-key-genes expression in validation cohort. Figure S13: MRPL11 and COX8A could promote ICC stemness features in a MAT2A-dependent manner. Figure S14: Pluripotent transcription factors were analyzed in RBE cell lines. Figure S15: The Wnt pathway activity and methionine metabolites in CSC and non-CSC. Figure S16: 4-key-genes could enhance EMT in ICC cells. Figure S17: The tumor growth curves of each group were summarized. Figure S18: The ROC curve of our nomogram for survival prediction in primary and validation cohort. Figure S19: Dietary methionine restriction for ICC patients with adjuvant TACE in validation cohort. Figure S20: Methionine therapy for ICC patients with adjuvant TACE in primary and validation cohort.

**Additional file 2** Table S1: Primers sequences for the detected genes. Table S2: Clinicopathologic characteristics. Table S3: Independent risk factors predicting OS for ICC patients with adjuvant TACE. Table S4: The clinicopathologic characteristics of subgroups stratified by the nomogram.

## Data Availability

All data generated or analyzed during this study are available from the corresponding author on reasonable request.

## Abbreviations

CSCs: Cancer stem cells
ICC: Intrahepatic cholangiocarcinoma
TACE: Transarterial chemoembolization
SDHAF2: Succinate dehydrogenase complex assembly factor 2
MRPS34: Mitochondrial ribosomal protein S34
MRPL11: Mitochondrial ribosomal protein L11
COX8A: Cytochrome c oxidase subunit 8A
CA19-9: Carbohydrate antigen 19-9
OS: Overall survival
C-index: Concordance index
DEGs: Differentially expressed genes
FDR: False discovery rate
mRNAsi: MRNA expression-based stemness index
EREG-mRNAsi: EPIGENETICALLY regulated mRNAsi
WGCNA: Weighted gene co-expression network analysis
GS: Gene significance
MM: Module membership
EGF: Epidermal growth factor
bFGF: Basic fibroblast growth factor
CCK8: Cell counting Kit-8
5-FU: 5-Fluorouracil
CDDP: Cisplatin
TMA: Tissue microarray
IHC: Immunohistochemistry
