# Supplementary material for: A gene signature is critical for intrahepatic cholangiocarcinoma stem cell self-renewal and chemotherapeutic response
Source: Stem Cell Res Ther. 2022 Jul 15;13:292. doi: 10.1186/s13287-022-02988-9 (PMC9284797; doi:10.1186/s13287-022-02988-9)
Supplement: Supplementary file 1 — Additional file 1 Figure S1: mRNAsi expression and associated key genes in ICC. Figure S2: Different soft threshold was analyzed to ensure a scale-free network. Figure S3: The correlation of 4-key-genes in non-CSC. Figure S4: The stemness and methionine cycle activity in ICC cell line. Figure S5: MRPL11 and COX8A are associated with stemness maintenance of cholangiocarcinoma CSCs. Figure S6: Pluripotent transcription factors were analyzed in various conditions. Figure S7: Flow cytometry analysis showed the overexpression of 4-key-genes could increase CD133+/CD44+ population in RBE cell. Figure S8: 4-key-genes level in CSC and non-CSC. Figure S9: The involvement of 4-key-genes in tumor proliferation. Figure S10: Cell viability was analyzed in 4-key-genes depletion and overexpression after treatment with 5-Fu. Figure S11: ICC with adjuvant TACE tends to recurrence when having high transcriptional level of the 4-key-genes. Figure S12: Kaplan–Meier analysis of the overall survival and cumulative recurrence in correlation with 4-key-genes expression in validation cohort. Figure S13: MRPL11 and COX8A could promote ICC stemness features in a MAT2A-dependent manner. Figure S14: Pluripotent transcription factors were analyzed in RBE cell lines. Figure S15: The Wnt pathway activity and methionine metabolites in CSC and non-CSC. Figure S16: 4-key-genes could enhance EMT in ICC cells. Figure S17: The tumor growth curves of each group were summarized. Figure S18: The ROC curve of our nomogram for survival prediction in primary and validation cohort. Figure S19: Dietary methionine restriction for ICC patients with adjuvant TACE in validation cohort. Figure S20: Methionine therapy for ICC patients with adjuvant TACE in primary and validation cohort. [file 13287_2022_2988_MOESM1_ESM.docx]

**Additional file 1: Figures**


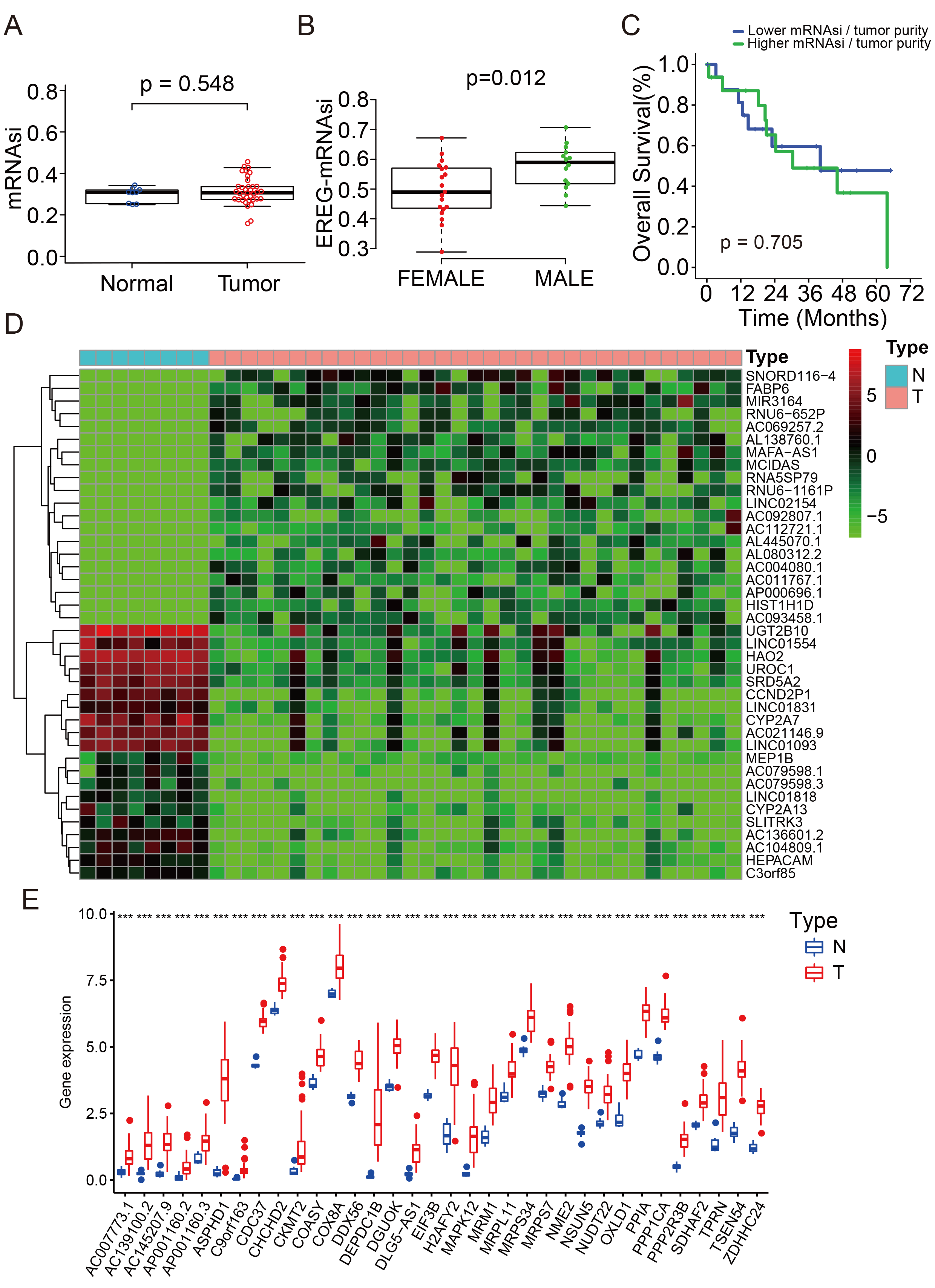


**Figure S1. mRNAsi expression and associated key genes in ICC.**

(A) Differences of mRNAsi between ICC (n = 36) and normal (n = 9) tissues in TCGA database. (B) Comparison of EREG-mRNAsi in male and female. (C) Kaplan-Meier analysis of the overall survival of ICC patients in correlation with corrected mRNAsi expression in TCGA database. (D) Heatmap of the first 20 upregulated key genes and the first 20 downregulated key genes in ICC tissues; Red represents high expression, and green indicates low expression. (E) Boxplot of the key genes in orange module.


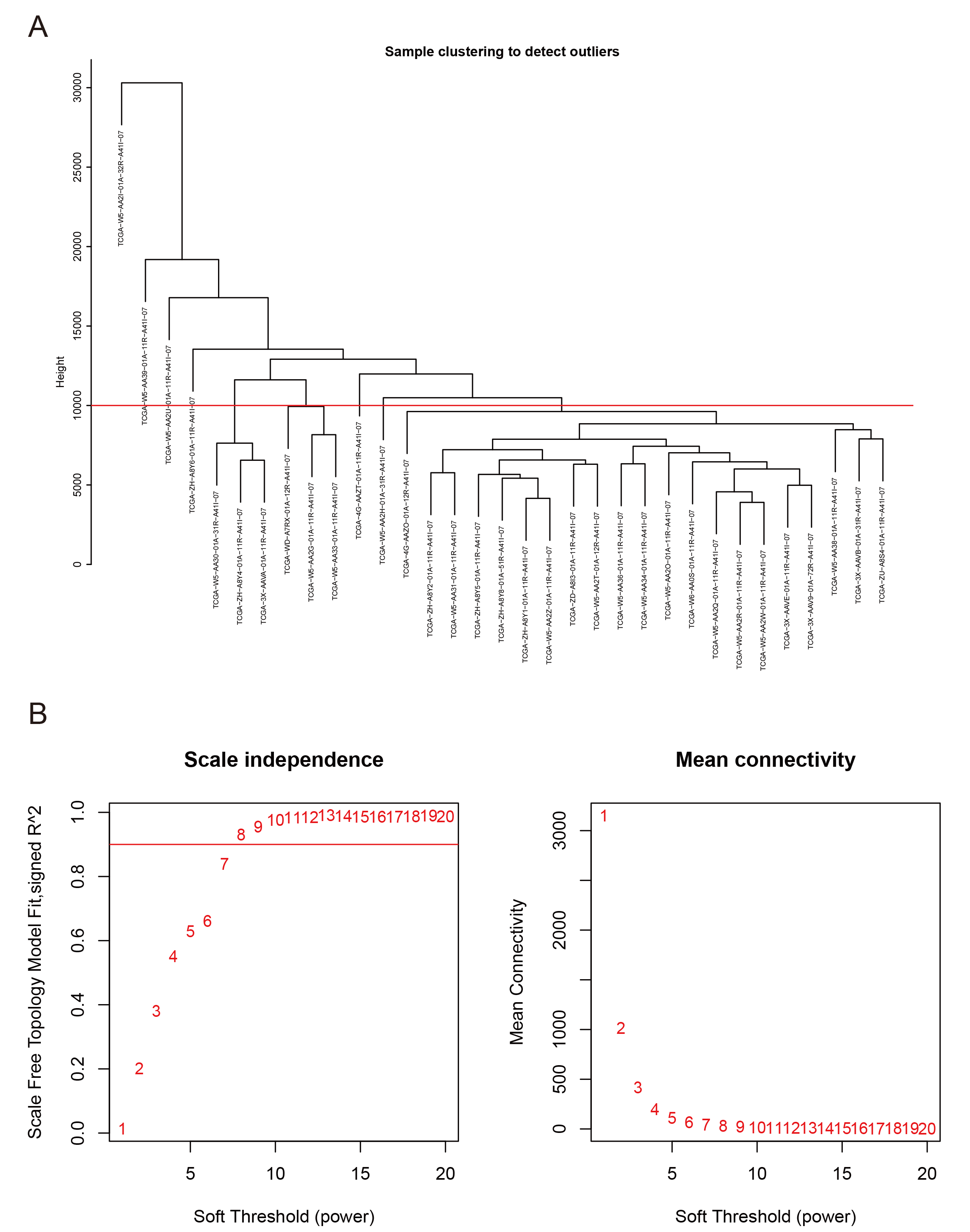


**Figure S2. Different soft threshold was analyzed to ensure a scale-free network.**

(A) Sample clustering to detect outliers. (B) Assessment of network topology for different soft-thresholding powers in scale independence and mean connectivity.


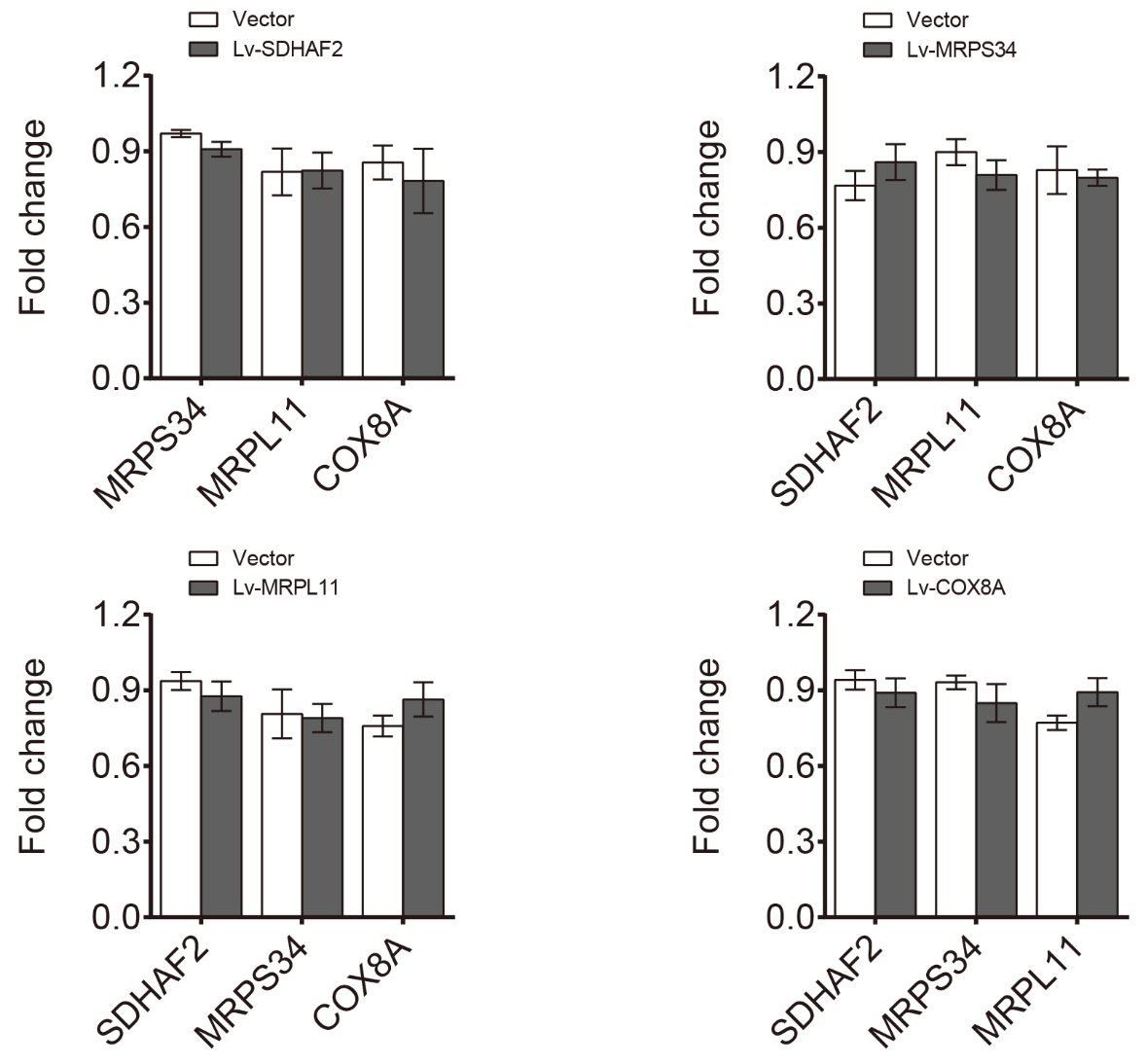


**Figure S3. The correlation of 4-key-genes in non-CSC.**

Non-CSC were isolated from ICC cells then 4-key-genes were overexpressed separately, their mRNA level were analyzed by qRT-PCR.


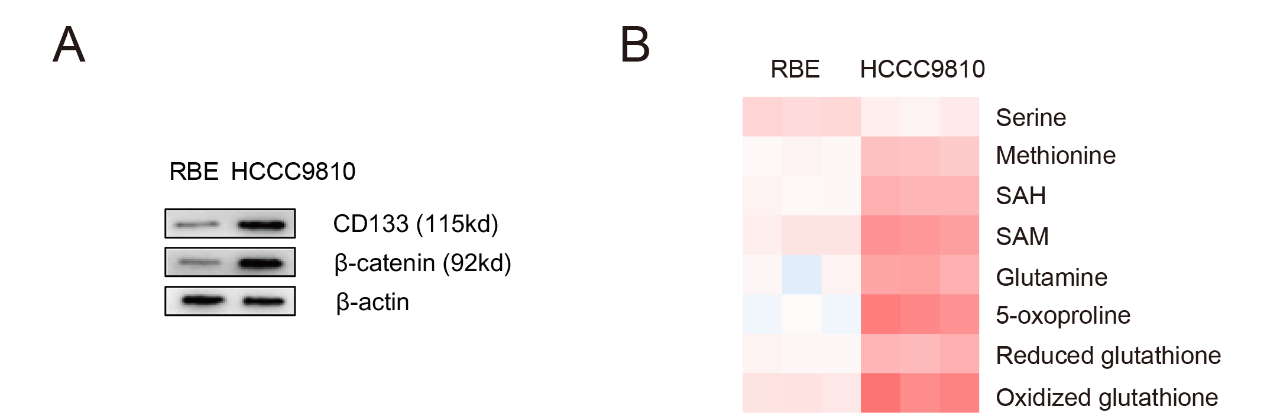


**Figure S4. The stemness and methionine cycle activity in ICC cell line.**

(A-B) The stemness markers (A) and methionine metabolites (B) in RBE and HCCC9810 cell lines.


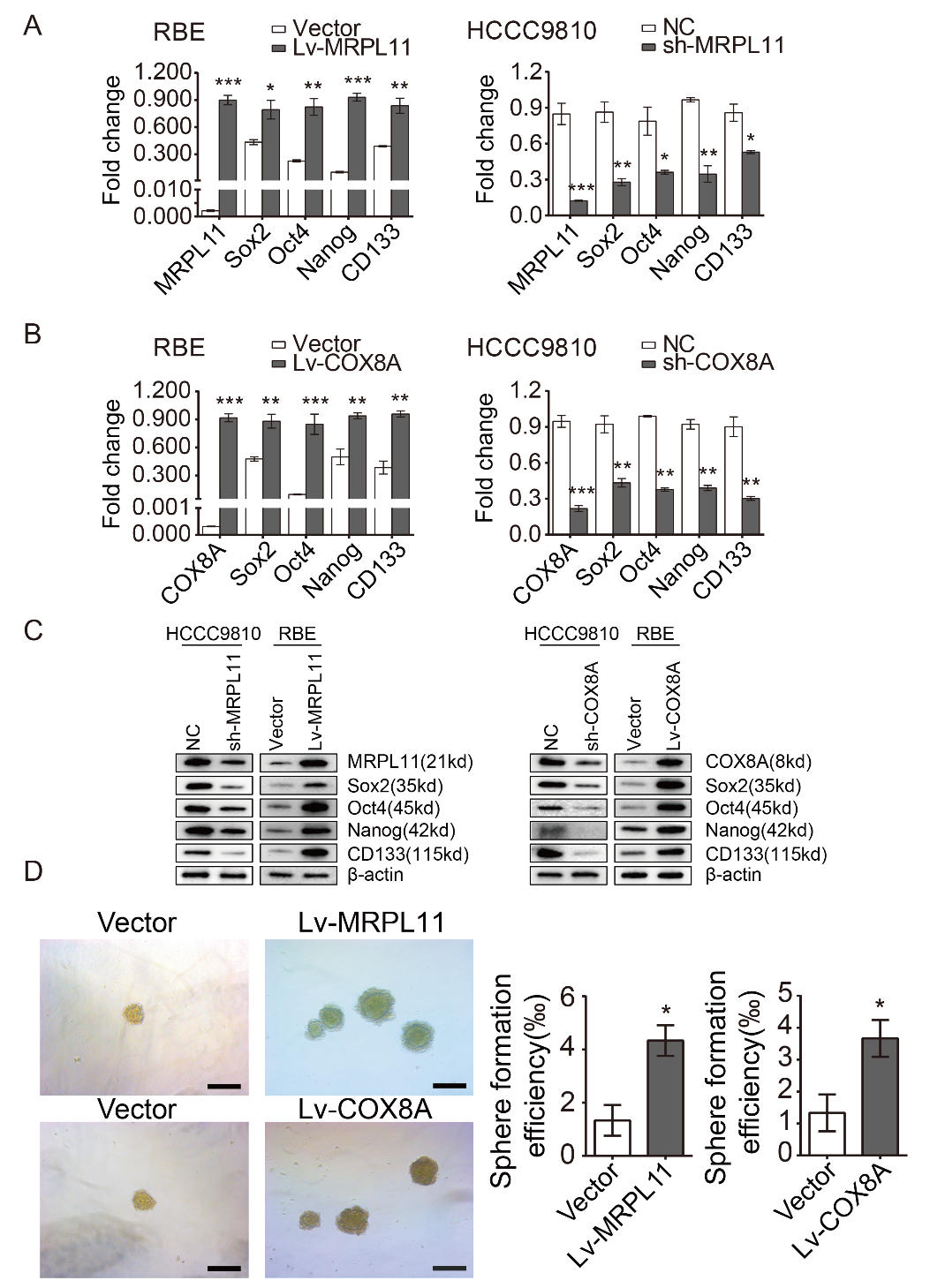


**Figure S5. MRPL11 and COX8A are associated with stemness maintenance of cholangiocarcinoma CSCs.**

(A-C) Pluripotent transcription factors were analyzed in MRPL11 (and COX8A) depletion or overexpression cells by qRT-PCR (A-B) and western blot (C). (D) MRPL11 or COX8A overexpression caused an enhanced oncosphere-forming capacity in RBE cells; the right panel represents statistical results as means ± SD; Scale bar, 100 μm. (*p < 0.05, **p < 0.01, ***p < 0.001)

**
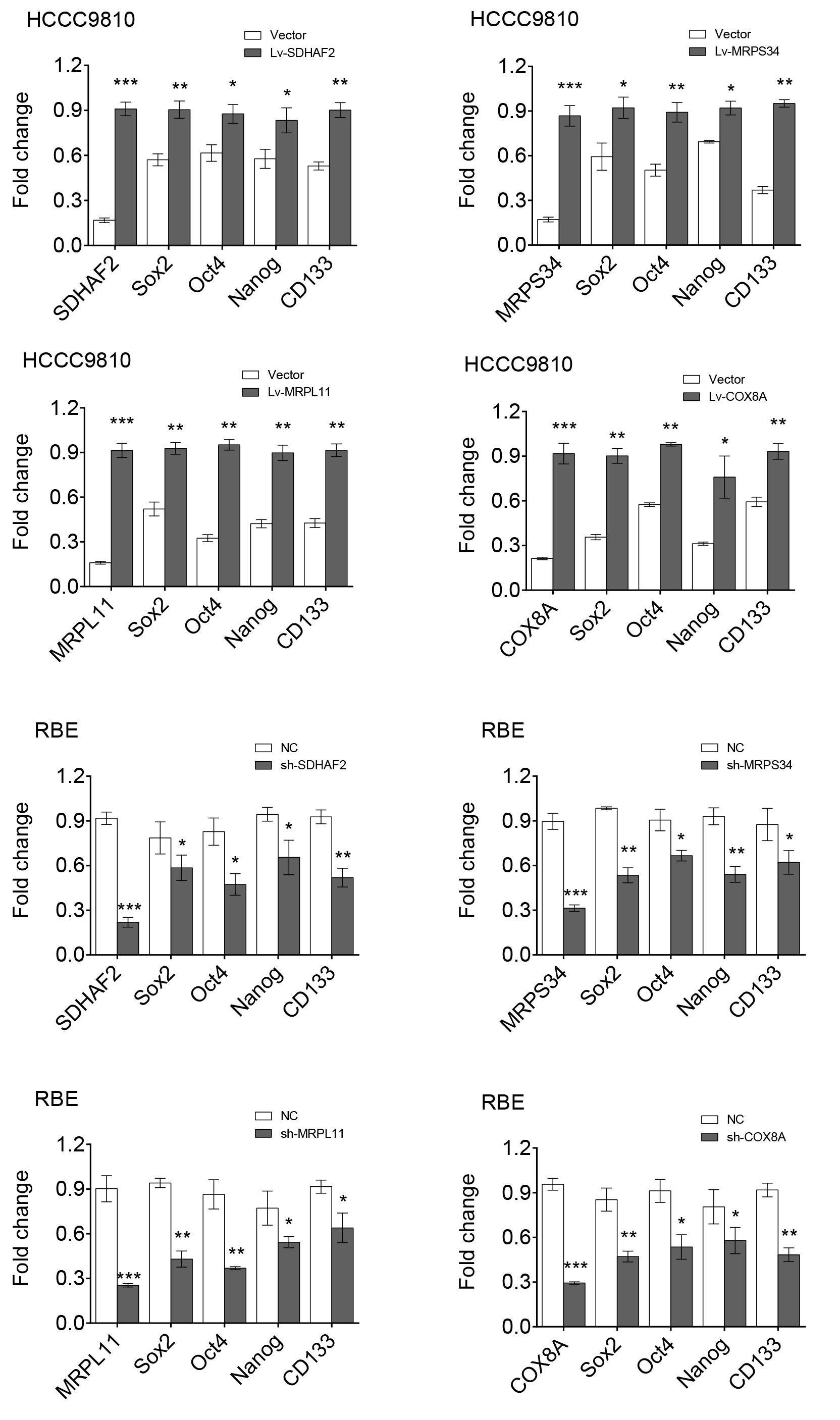
**

**Figure S6. Pluripotent transcription factors were analyzed in various conditions.**


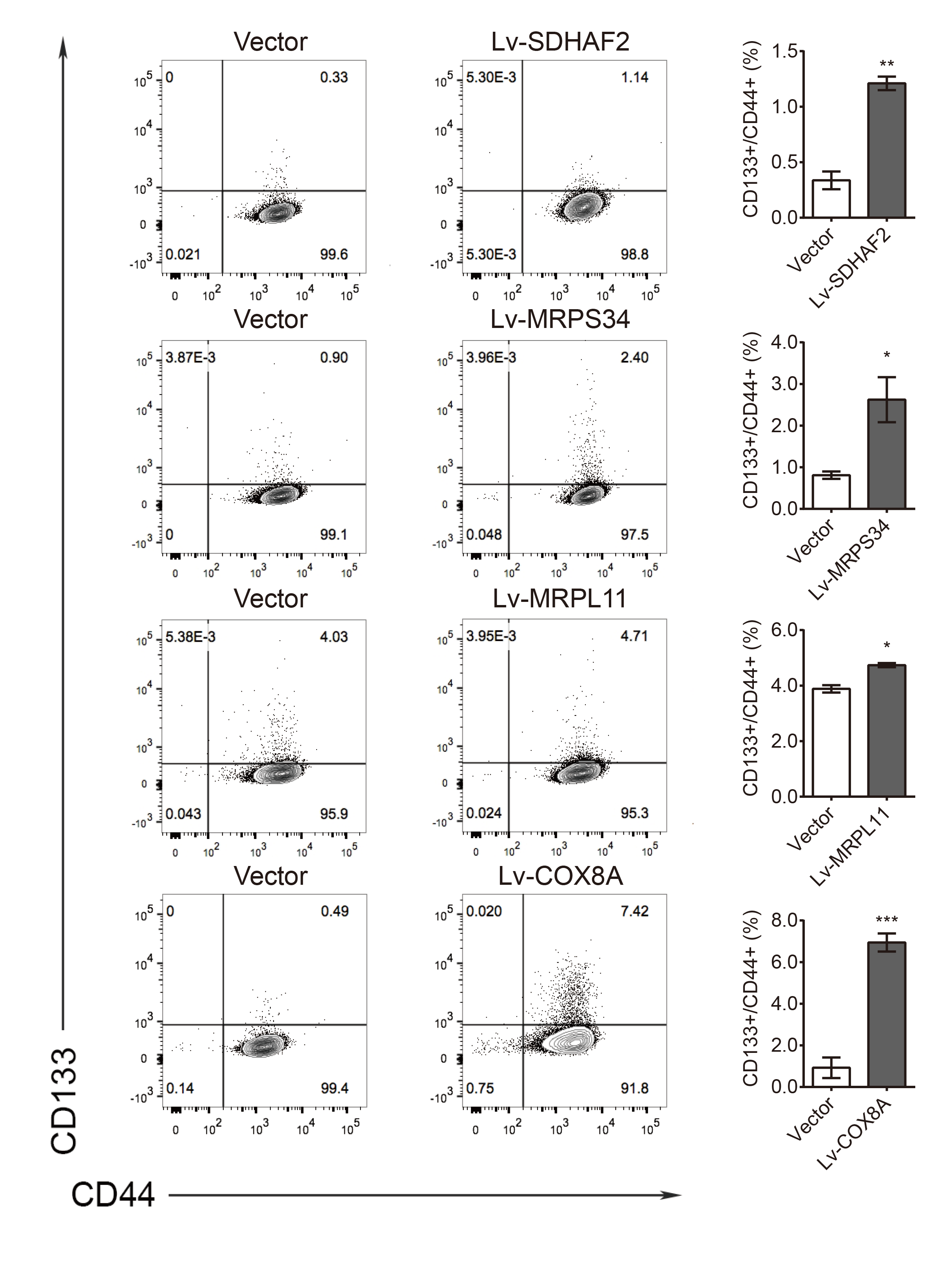


**Figure S7. Flow cytometry analysis showed the overexpression of 4-key-genes could increase CD133^+^/CD44^+^ population in RBE cell.**


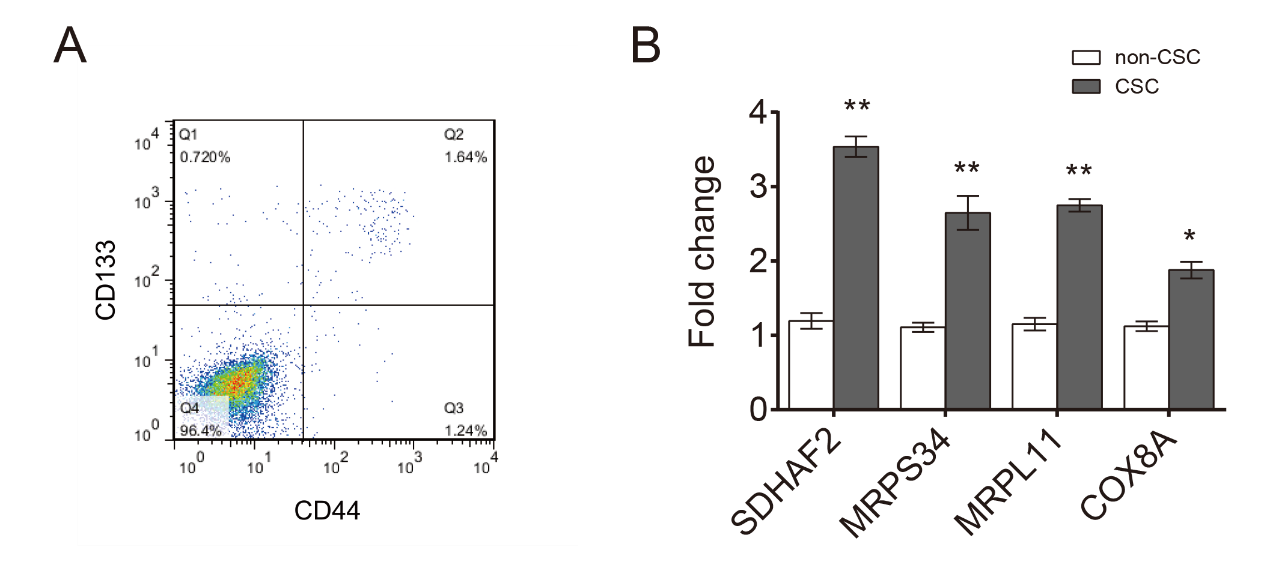


**Figure S8. 4-key-genes level in CSC and non-CSC.**

(A) CSC were isolated from primary ICC cells by flow sorting (CD133^+^CD44^+^), while the rest cells were defined as non-CSC. (B) The mRNA level of 4-key-genes in CSC and non-CSC.

**
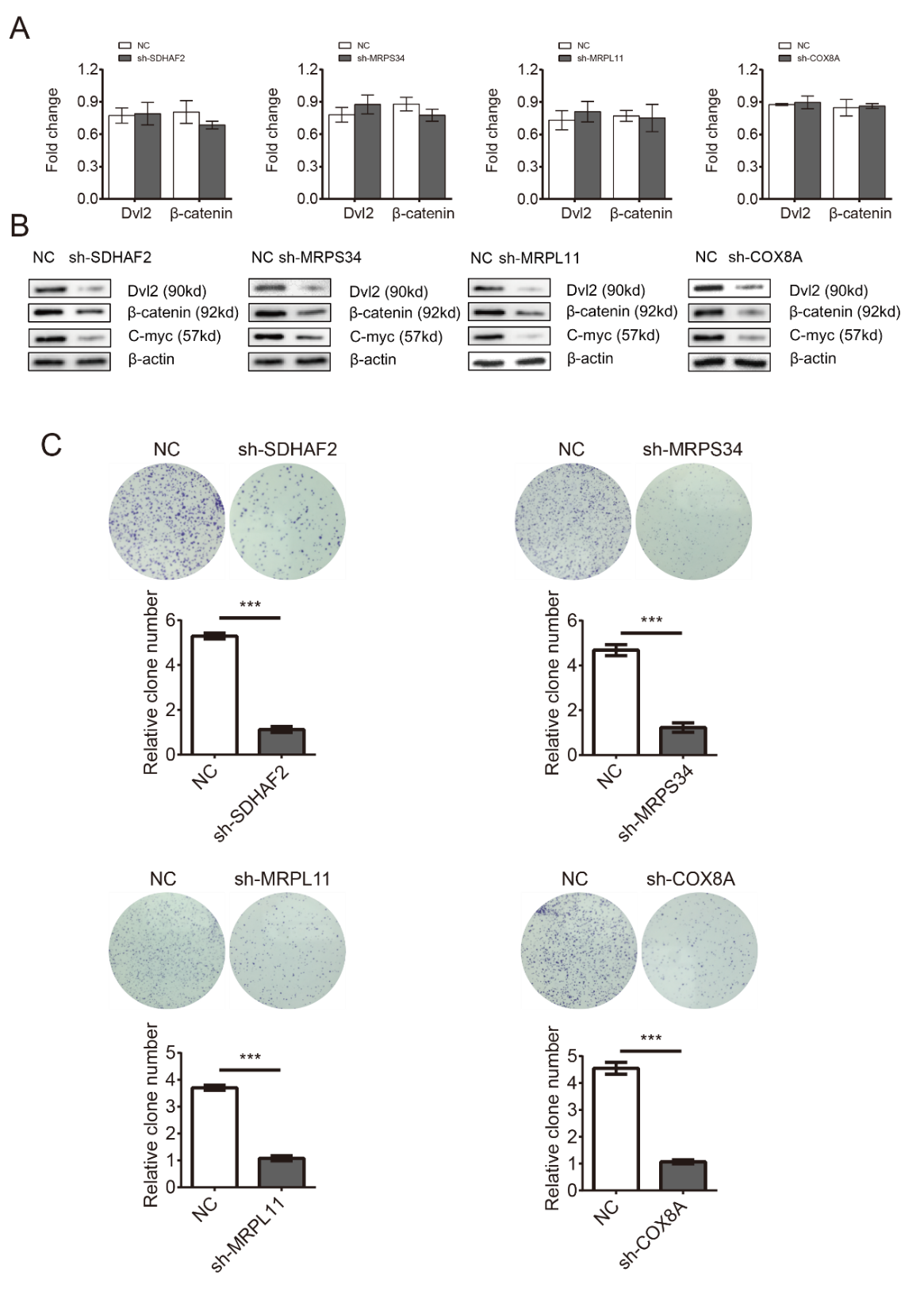
**

**Figure S9. The involvement of 4-key-genes in tumor proliferation.**

(A) mRNA level of Dvl2 and β-catenin in CSC with knockdown of 4-key-genes. (B) Wnt pathway activity in CSC with knockdown of 4-key-genes. (C) Representative images and quantification of colony formation ability in CSC with or without knockdown of 4-key-genes.


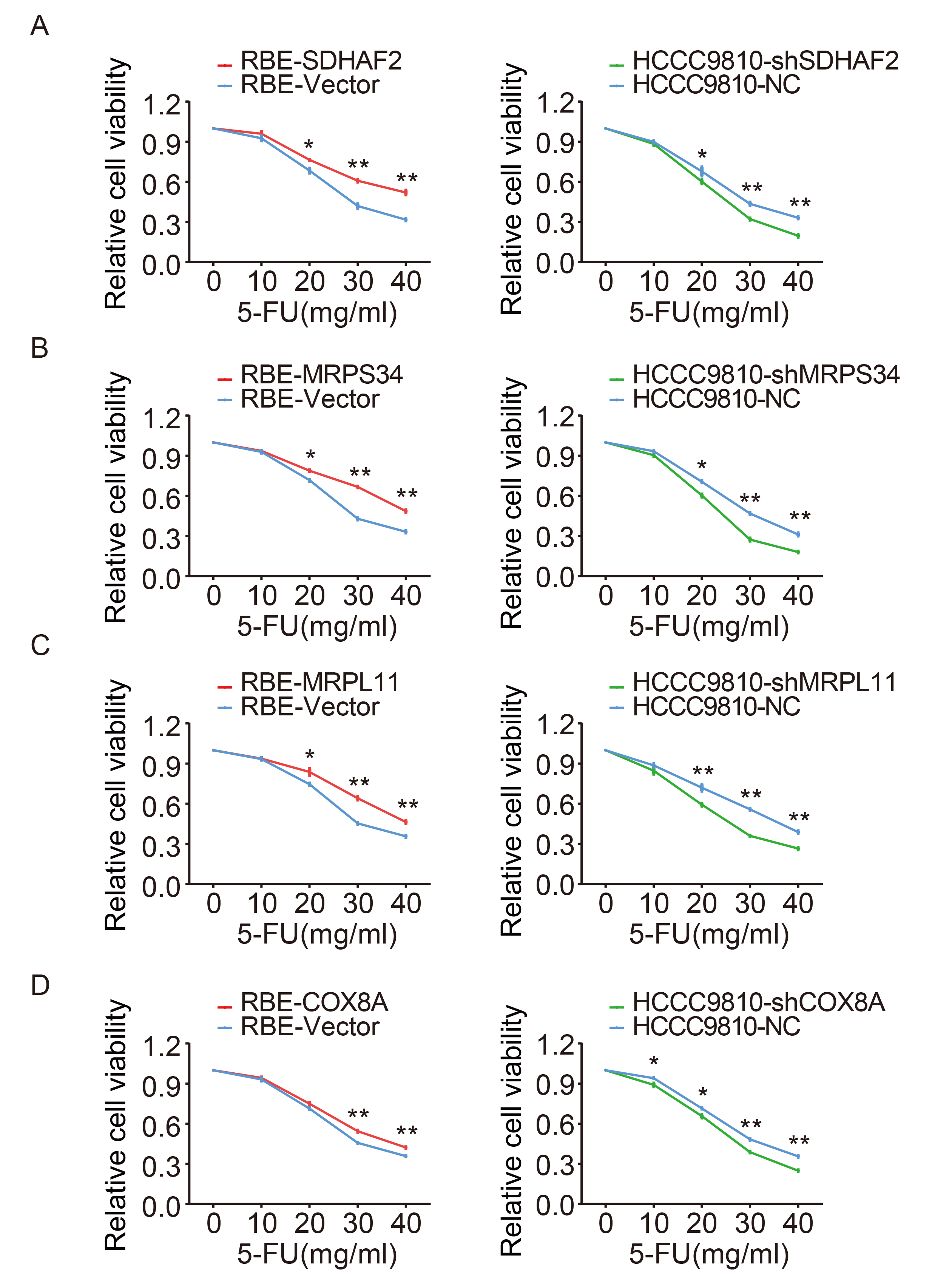


**Figure S10. Cell viability were analyzed in 4-key-genes depletion and overexpression after treatment with 5-Fu.**


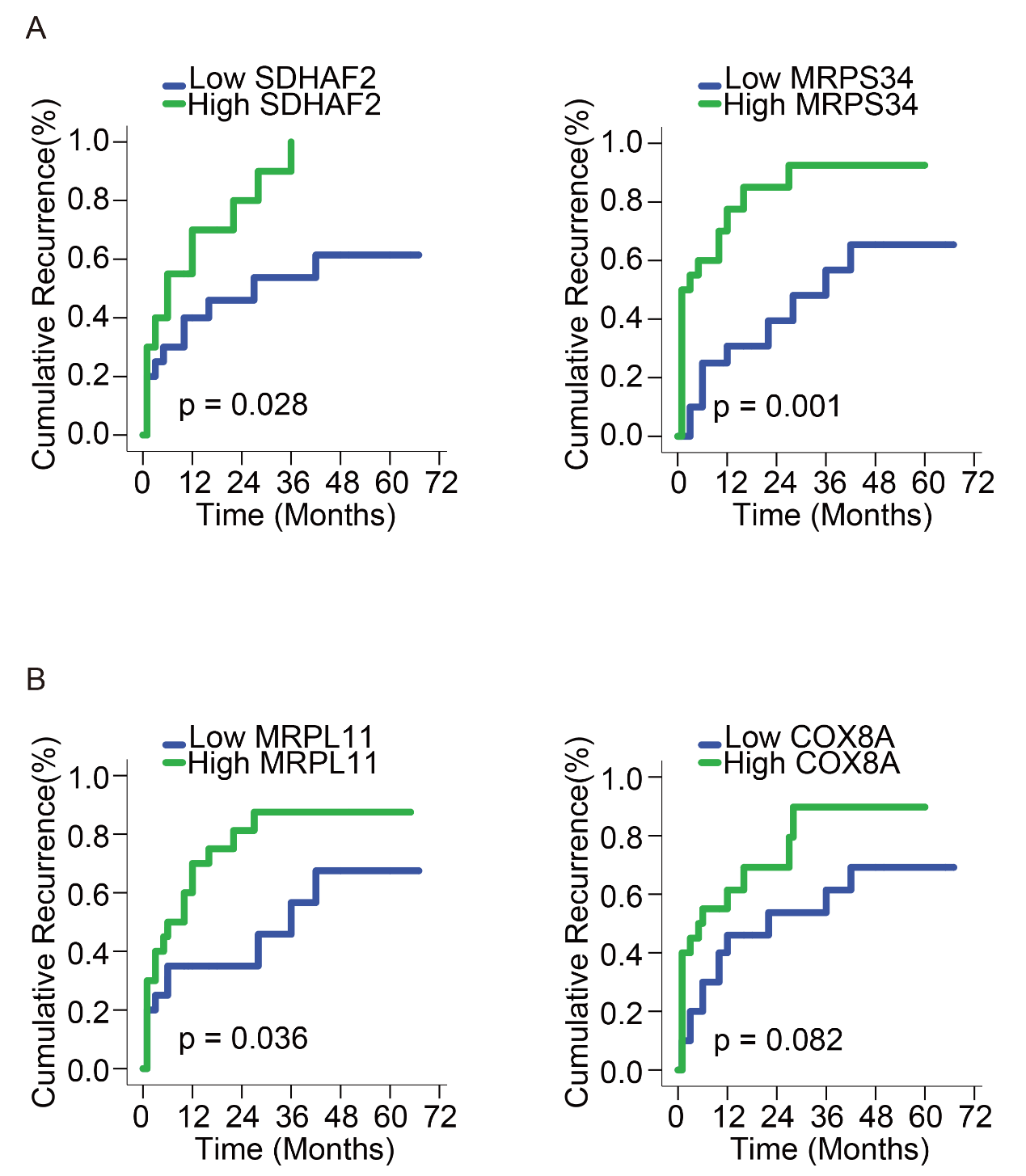


**Figure S11. ICC with adjuvant TACE tend to recurrence when having high transcriptional level of the 4-key-genes.**


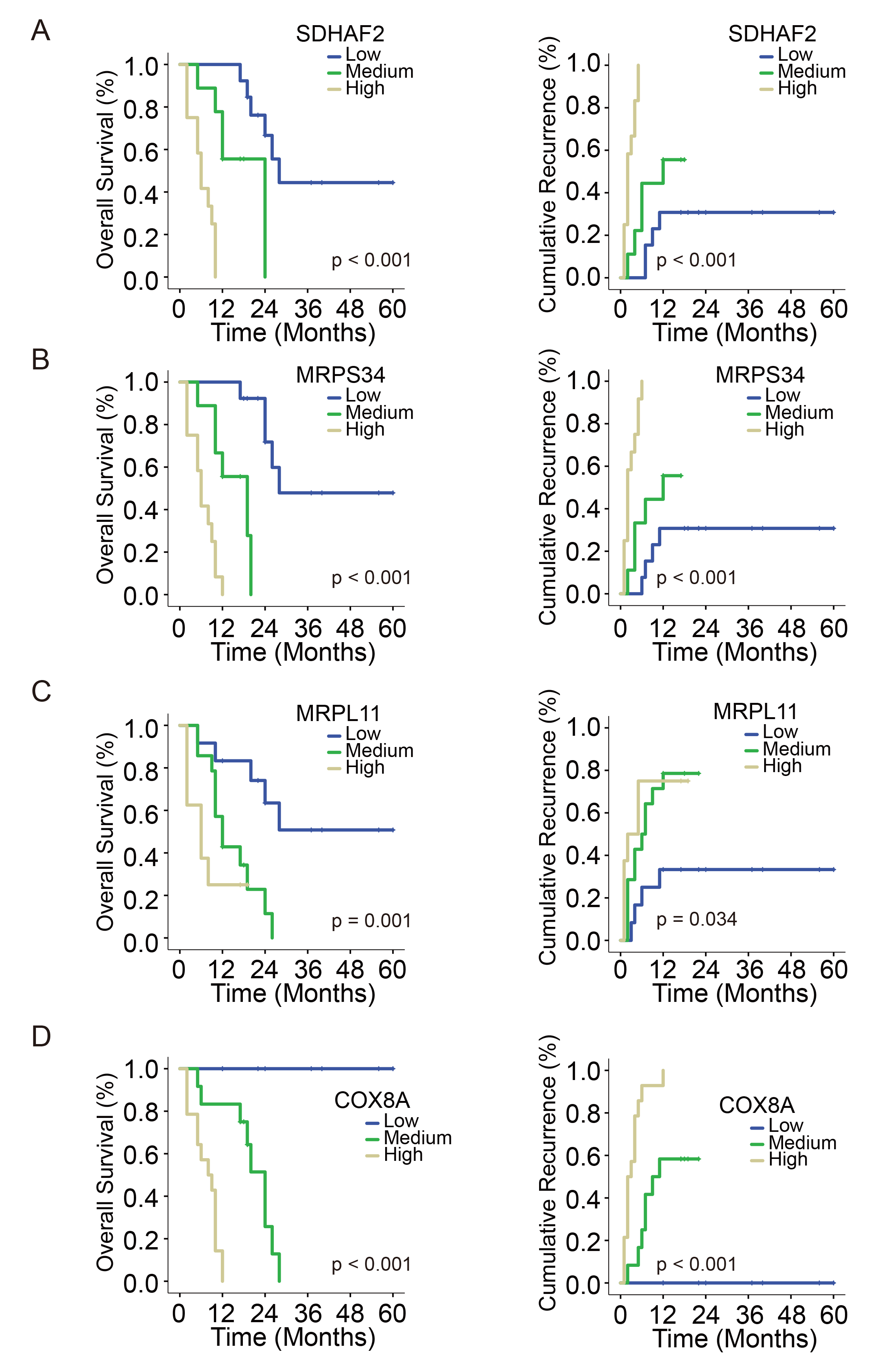


**Figure S12. Kaplan-Meier analysis of the overall survival and cumulative recurrence in correlation with 4-key-genes expression in validation cohort.**

**
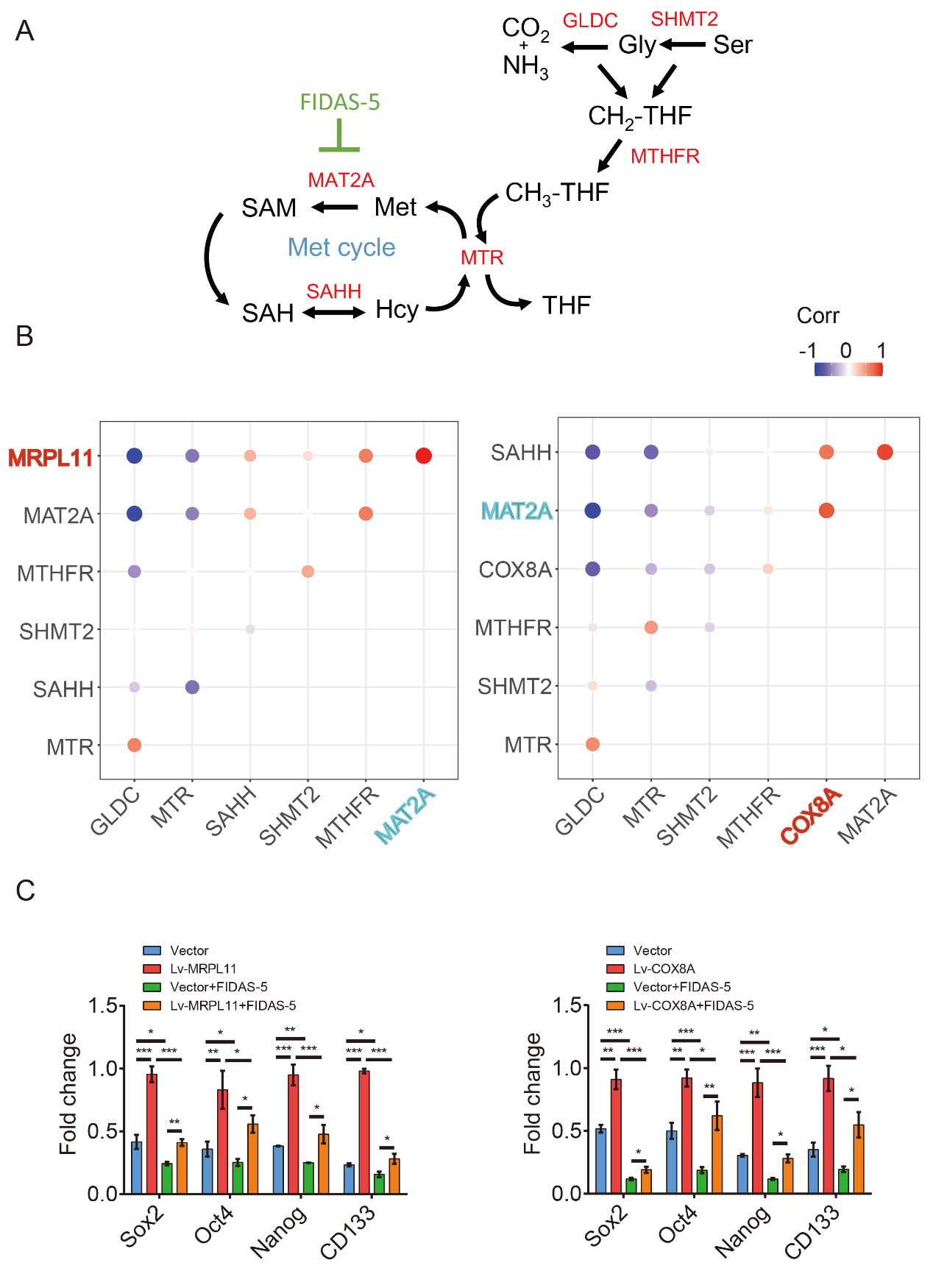
**

**Figure S13. MRPL11 and COX8A could promote ICC stemness features in a MAT2A dependent manner.**

(A) Schematic of the methionine cycle pathways. (B) The correlation between MRPL11 (or COX8A) and key enzymes of methionine cycle in primary ICC cells. (C) Vector and Lv-MRPL11 HCCC9810 cells (or Lv-COX8A HCCC9810 cells) were treated with or without FIDAS-5, then the expression of pluripotent transcription factors were analyzed by qRT-PCR. (*p < 0.05, **p < 0.01, ***p < 0.001)

**
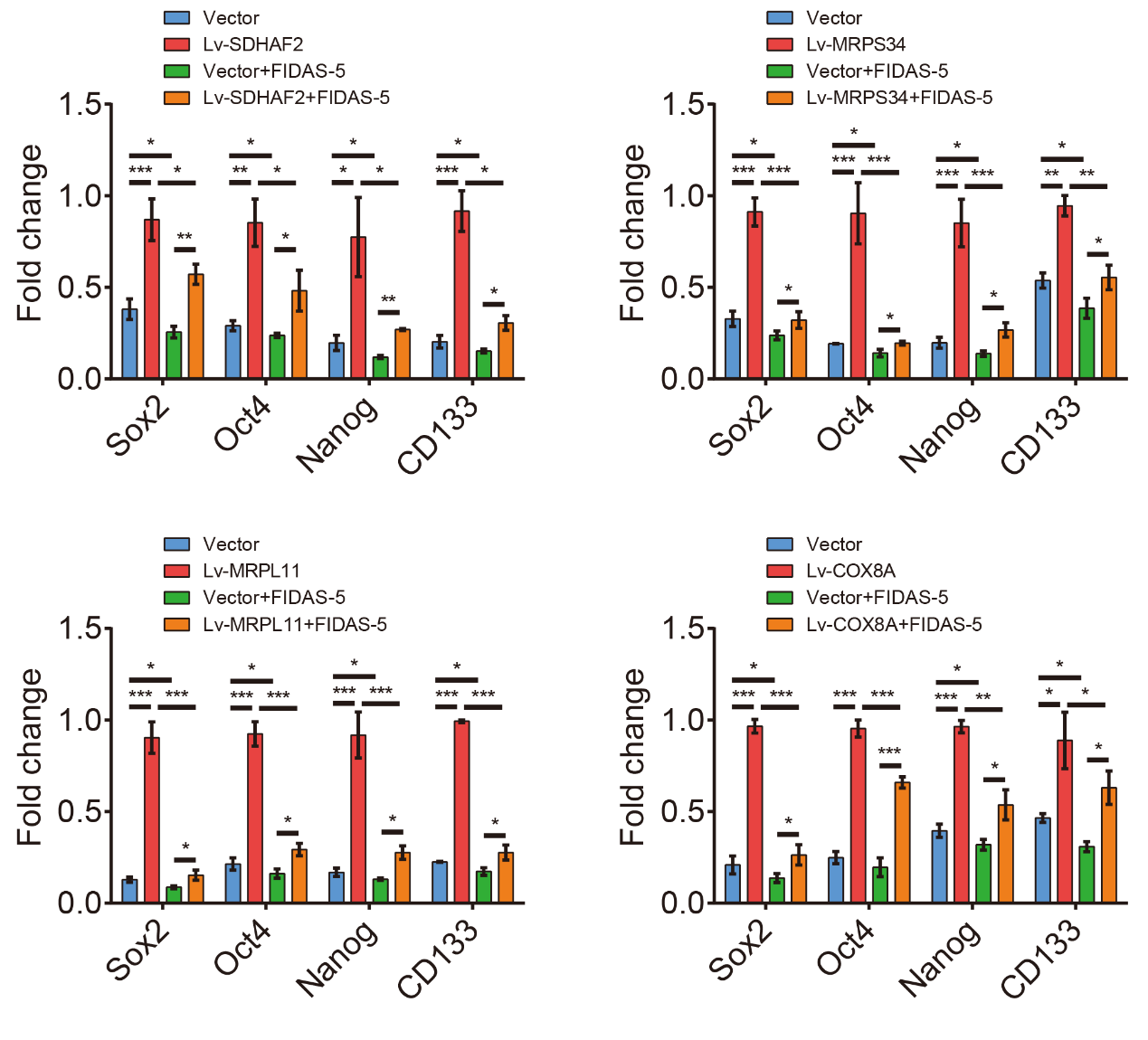
**

**Figure S14. Pluripotent transcription factors were analyzed in RBE cell lines.**


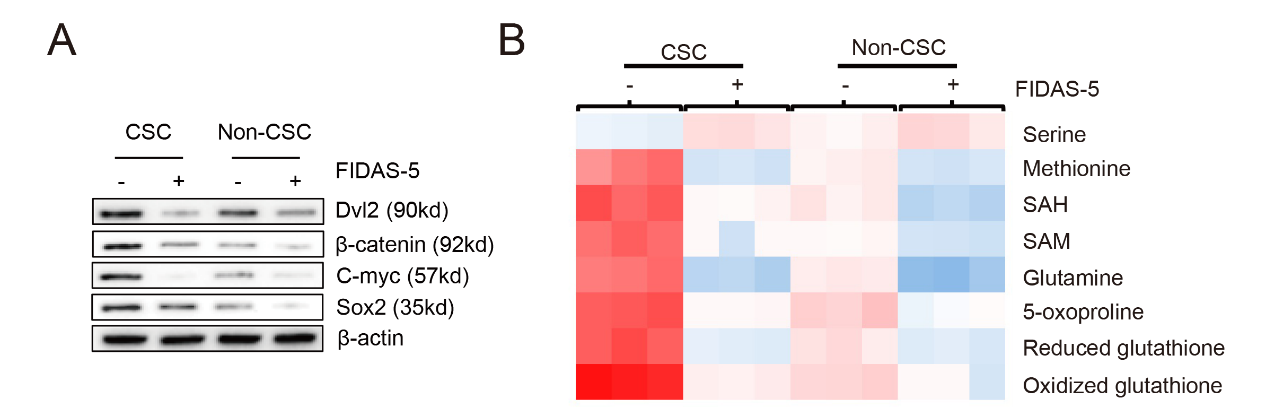


**Figure S15. The Wnt pathway activity and methionine metabolites in CSC and non-CSC.**


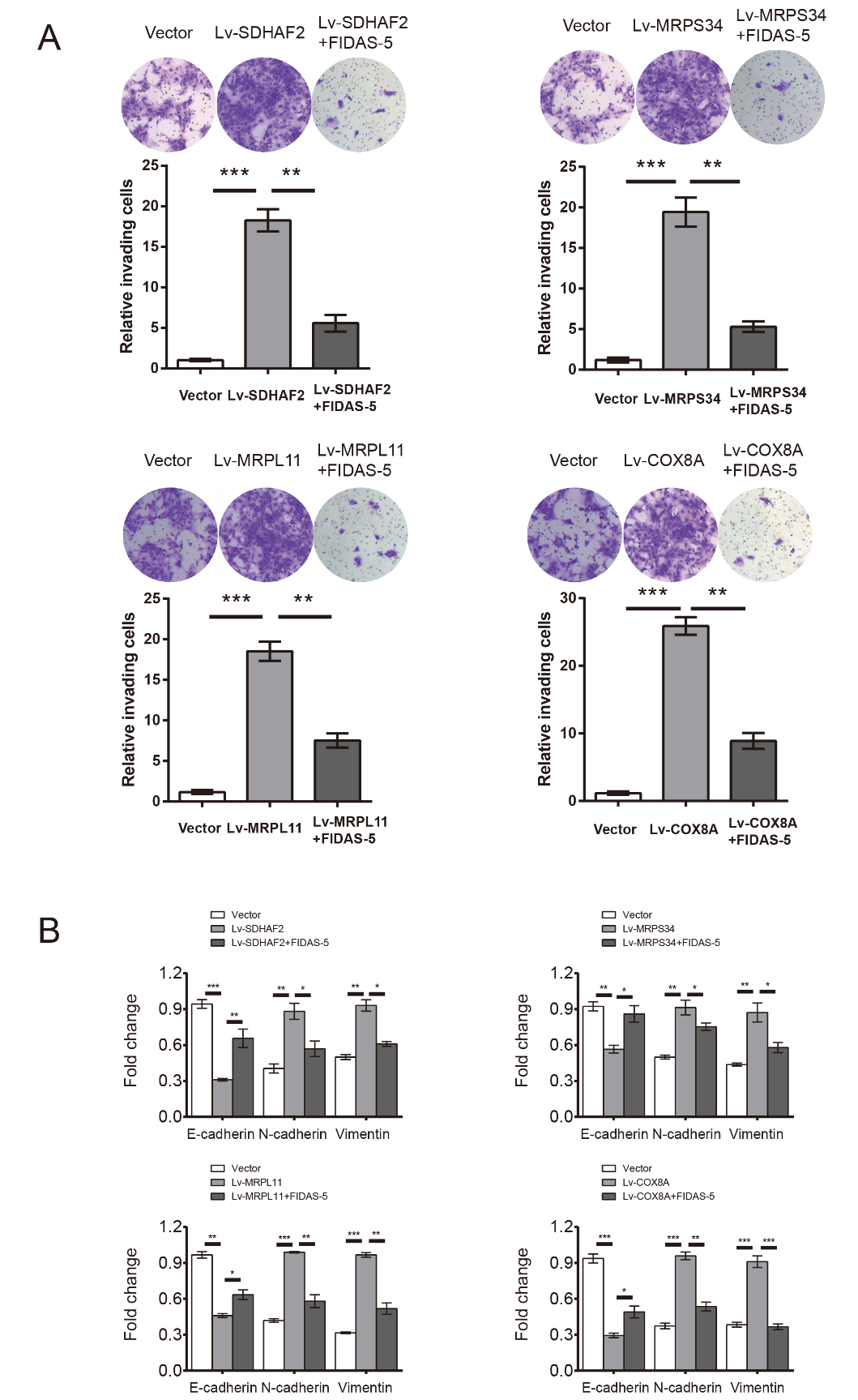


**Figure S16. 4-key-genes could enhance EMT in ICC cells.**

(A) The transwell assay of RBE cell line in overexpression of 4-key-genes with or without methionine cycle inhibition. (B) The mRNA levels of EMT markers (E-cadherin, N-cadherin and Vimentin) in RBE cell line.


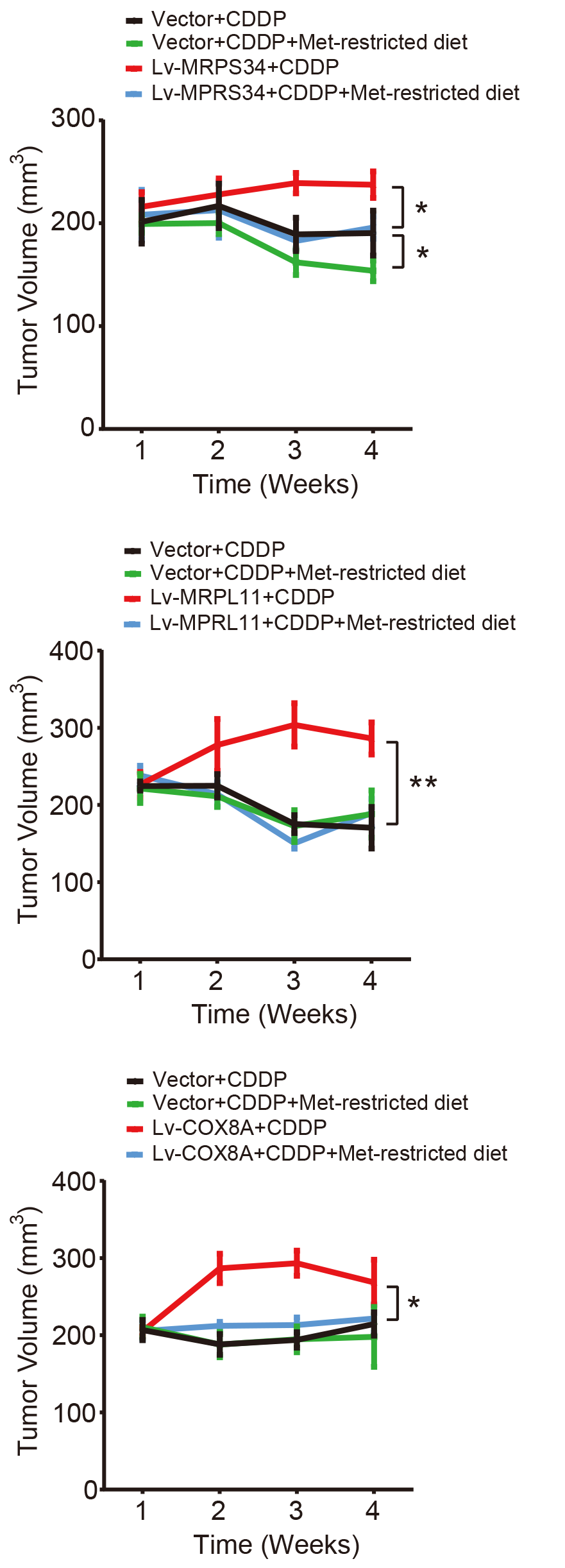


**Figure S17. The tumor growth curves of each group were summarized.**


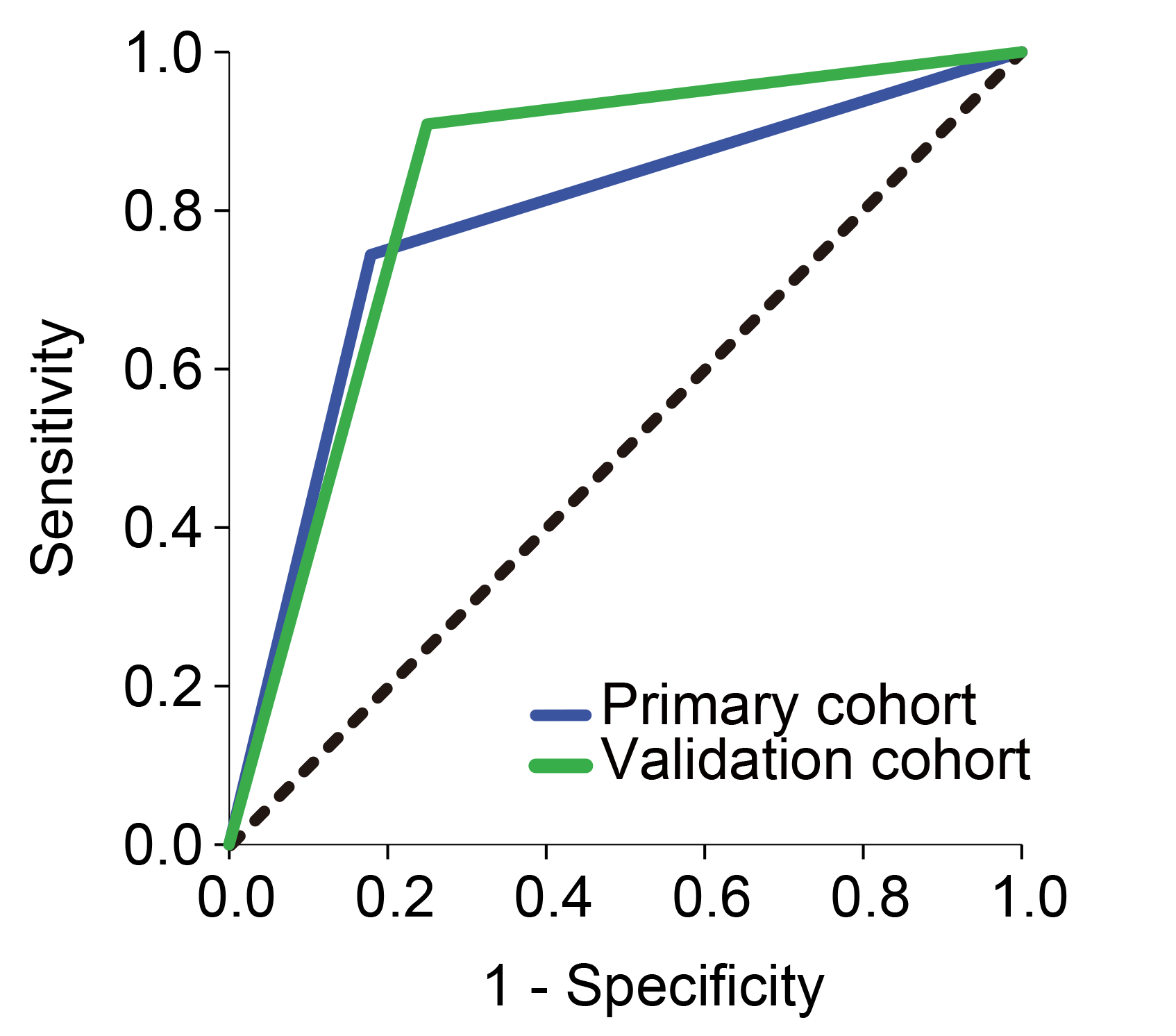


**Figure S18. The ROC curve of our nomogram for survival prediction in primary and validation cohort.**


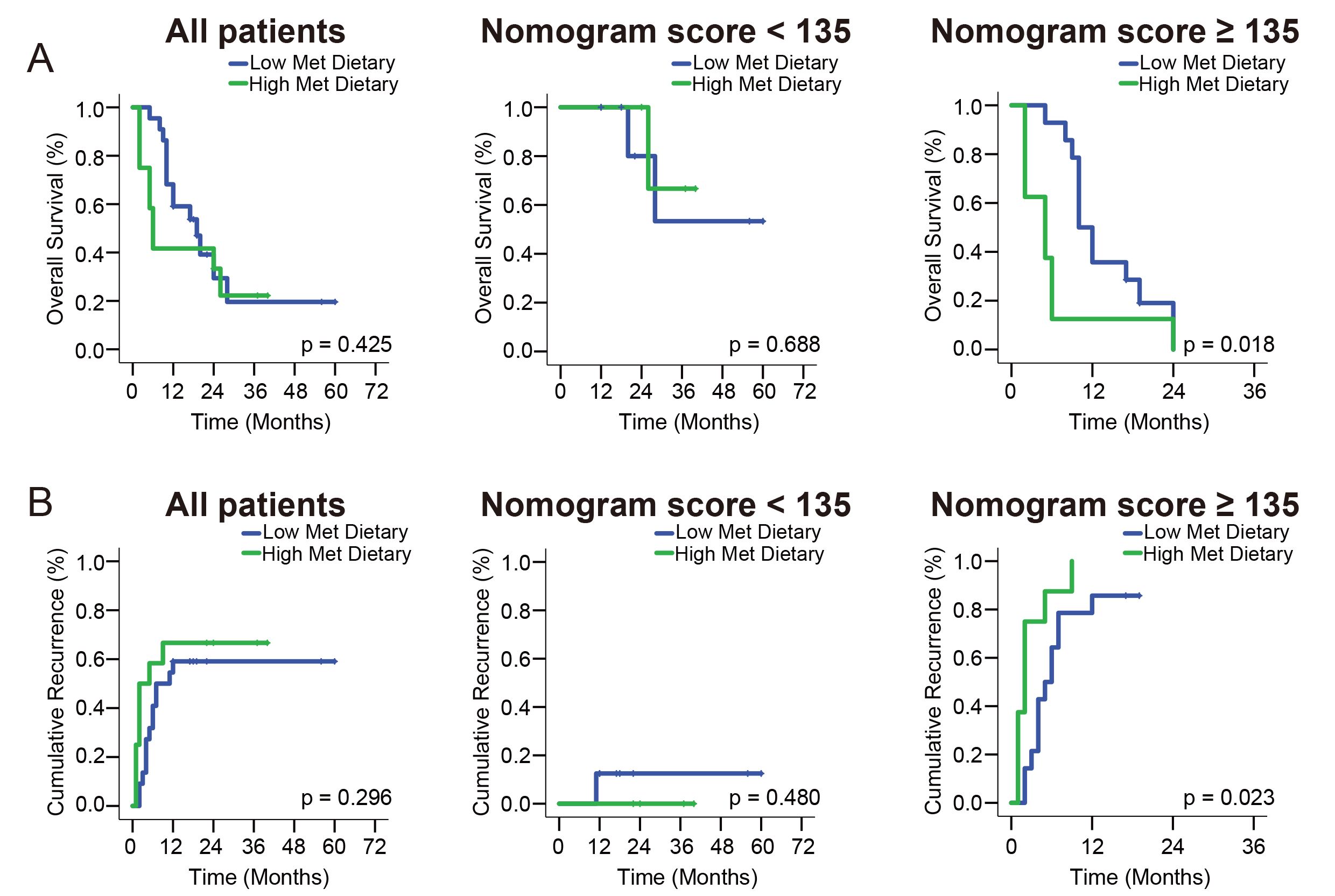


**Figure S19. Dietary methionine restriction for ICC patients with adjuvant TACE in validation cohort.**

(A) Comparison of overall survival rates between patients with low and high methionine dietary in validation cohort. (B) Comparison of cumulative recurrence rates between patients with low and high methionine dietary in validation cohort. (All patients in validation cohort, n = 34; Patients with nomogram score < 135 in validation cohort, n = 12; Patients with nomogram score ≥ 135 in validation cohort, n = 22)


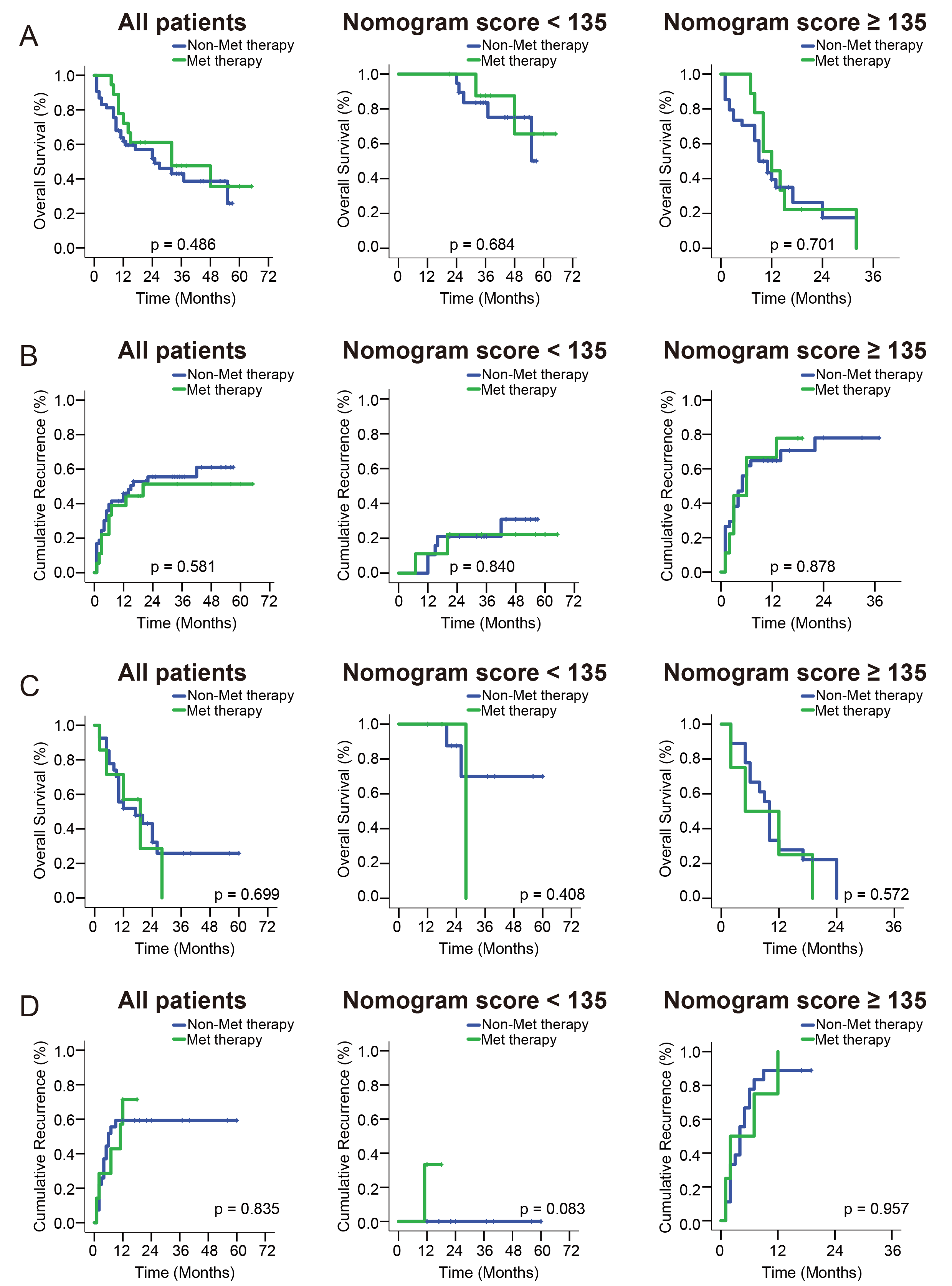


**Figure S20. Methionine therapy for ICC patients with adjuvant TACE in primary and validation cohort.**

(A) Comparison of overall survival rates between patients with or without methionine therapy in primary cohort. (B) Comparison of cumulative recurrence rates between patients with or without methionine therapy in primary cohort. (C) Comparison of overall survival rates between patients with or without methionine therapy in validation cohort. (D) Comparison of cumulative recurrence rates between patients with or without methionine therapy in validation cohort.
