# Supplementary material for: A gene signature is critical for intrahepatic cholangiocarcinoma stem cell self-renewal and chemotherapeutic response
Source: Stem Cell Res Ther. 2022 Jul 15;13:292. doi: 10.1186/s13287-022-02988-9 (PMC9284797; doi:10.1186/s13287-022-02988-9)
Supplement: Supplementary file 2 — Additional file 2 Table S1: Primers sequences for the detected genes. Table S2: Clinicopathologic characteristics. Table S3: Independent risk factors predicting OS for ICC patients with adjuvant TACE. Table S4: The clinicopathologic characteristics of subgroups stratified by the nomogram. [file 13287_2022_2988_MOESM2_ESM.docx]

**Additional file 2: Tables**

| **Table S1. Primers sequences for the detected genes** | |
| --- | --- |
| SDHAF2 forward | 5’-TTCTCGACTTCGTCGCTGATG-3’ |
| SDHAF2 reverse | 5’-CACCTCTGTAGAAGCGTCTGA-3’ |
| MRPS34 forward | 5’-CTCCGGGCCATGATTATCGC-3’ |
| MRPS34 reverse | 5’-CCATGCGTATCCTCTGCACAT-3’ |
| MRPL11 forward | 5’-GGCGTTTCCATCAACCAGTTT-3’ |
| MRPL11 reverse | 5’-TGCGGGCAATCTCATACACAT-3’ |
| COX8A forward | 5’-GCCAAGATCCATTCGTTGCC-3’ |
| COX8A reverse | 5’-CTCTGGCCTCCTGTAGGTCT-3’ |
| Sox2 forward | 5’-CCATGCAGGTTGACACCGTTG-3’ |
| Sox2 reverse | 5’-TCGGCAGACTGATTCAAATAATACAG-3’ |
| Oct4 forward | 5’-GGGCTCTCCCATGCATTCAAAC-3’ |
| Oct4 reverse | 5’-CACCTTCCCTCCAACCAGTTGC-3’ |
| Nanog forward | 5’-AAGGTCCCGGTCAAGAAACAG-3’ |
| Nanog reverse | 5’-CTTCTGCGTCACACCATTGC-3’ |
| CD133 forward | 5’-AGTCGGAAACTGGCAGATAGC-3’ |
| CD133 reverse | 5’-GGTAGTGTTGTACTGGGCCAAT-3’ |
| β-actin forward | 5’-GGGAAATCGTGCGTGACATTAAG-3’ |
| β-actin reverse | 5’-TGTGTTGGCGTACAGGTCTTTG-3’ |

| **Table S2. Clinicopathologic characteristics** | | | | | | |
| --- | --- | --- | --- | --- | --- | --- |
| Variable |  | Patients | | | | |
|  |  | Primary cohort  (71) |  | Validation cohort  (34) |  | p value |
| Age, yrs |  | 55(25-82) |  | 54(27-77) |  | 0.137 |
| Sex, M:F |  | 48:23 |  | 25:9 |  | 0.537 |
| HBsAg, positive: negative |  | 35:36 |  | 20:14 |  | 0.360 |
| Platelet, 10^9^/L |  | 179(79-310) |  | 180(41-309) |  | 0.851 |
| Albumin, g/L |  | 40(22-58) |  | 39(22-49) |  | 0.808 |
| Total bilirubin, μmol/L |  | 14(4-79) |  | 14(5-328) |  | 0.144 |
| Prothrombin time, sec |  | 11(9-18) |  | 12(10-20) |  | 0.085 |
| AFP, ng/ml |  | 5(1-3000) |  | 4(1-3000) |  | 0.087 |
| CA19-9, U/ml |  | 63(1-10000) |  | 324 (13-10000) |  | 0.784 |
| Nodules, multiple: single |  | 23:48 |  | 14:20 |  | 0.378 |
| Encapsulation, complete: incomplete |  | 15:56 |  | 11:23 |  | 0.212 |
| Microvascular invasion, yes: no |  | 25:46 |  | 10:24 |  | 0.555 |
| Tumor size, cm |  | 5.0(1.0-15.0) |  | 6.0(1.2-10.0) |  | 0.151 |
| Lymph node metastasis, yes: no |  | 28:43 |  | 8:26 |  | 0.108 |
| Differentiation, III-IV: I-II |  | 46:25 |  | 26:8 |  | 0.228 |
| TNM stage, III-IV: I-II |  | 38:33 |  | 15:19 |  | 0.367 |
| SDHAF2, high: medium: low |  | 16:28:27 |  | 12:9:13 |  | 0.286 |
| MRPS34, high: medium: low |  | 22:24:25 |  | 12:9:13 |  | 0.747 |
| MRPL11, high: medium: low |  | 25:18:28 |  | 8:14:12 |  | 0.224 |
| COX8A, high: medium: low |  | 20:25:26 |  | 14:12:8 |  | 0.299 |
| Abbreviations: |  |  |  |  |  |  |
| AFP, alpha-fetoprotein; CA19-9, Carbohydrate Antigen 19-9; HBsAg, hepatitis B surface antigen. | | | | | | |

| **Table S3. Independent risk factors predicting OS for ICC patients with adjuvant TACE** | | | | | | | | | | |
| --- | --- | --- | --- | --- | --- | --- | --- | --- | --- | --- |
|  |  | **Univariate Analysis** | | |  | | **Multivariate Analysis** | | |  |
| **Variables** | **HR** | | **95% CI** | ***p-*value** | | **HR** | | **95% CI** | ***p*-value** | |
| Age, yrs | 1.01 | | 0.98-1.04 | 0.475 | |  | |  | NA | |
| Sex, M:F | 0.77 | | 0.39-1.50 | 0.440 | |  | |  | NA | |
| HBsAg, positive: negative | 0.88 | | 0.47-1.66 | 0.698 | |  | |  | NA | |
| Platelet,10^9^/L | 1.00 | | 0.99-1.01 | 0.453 | |  | |  | NA | |
| Albumin, g/L | 0.99 | | 0.98-1.01 | 0.347 | |  | |  | NA | |
| Total bilirubin, μmol/L | 1.01 | | 0.99-1.03 | 0.255 | |  | |  | NA | |
| Prothrombin time, sec | 1.01 | | 0.84-1.23 | 0.902 | |  | |  | NA | |
| AFP, ng/ml | 1.00 | | 0.99-1.00 | 0.660 | |  | |  | NA | |
| CA19-9, U/ml | 1.00 | | 1.00-1.01 | **<0.001** | | 1.01 | | 1.00-1.01 | **0.003** | |
| Nodules, multiple: single | 3.52 | | 1.84-6.73 | **<0.001** | |  | |  | NA | |
| Encapsulation, complete: incomplete | 2.78 | | 1.31-5.90 | **0.008** | |  | |  | NA | |
| Microvascular invasion, yes: no | 2.66 | | 1.39-5.08 | **0.003** | |  | |  | NA | |
| Tumor size, cm | 1.51 | | 1.34-1.71 | **<0.001** | | 1.21 | | 1.03-1.43 | **0.023** | |
| Lymph node metastasis, yes: no | 8.70 | | 4.30-17.59 | **<0.001** | | 6.97 | | 2.61-18.62 | **<0.001** | |
| Differentiation, III-IV: I-II | 1.11 | | 0.58-2.15 | 0.752 | |  | |  | NA | |
| SDHAF2, high: medium: low | 3.99 | | 2.54-6.26 | **<0.001** | | 2.26 | | 1.02-4.98 | **0.044** | |
| MRPS34, high: medium: low | 2.28 | | 1.53-3.40 | **<0.001** | |  | |  | NA | |
| MRPL11, high: medium: low | 2.76 | | 1.80-4.23 | **<0.001** | | 2.25 | | 1.29-3.92 | **0.004** | |
| COX8A, high: medium: low | 3.66 | | 2.38-5.64 | **<0.001** | | 3.71 | | 1.72-8.01 | **0.001** | |
| Abbreviations: |  | |  |  | |  | |  |  | |
| AFP, alpha-fetoprotein; CA19-9, Carbohydrate Antigen 19-9; HR, hazard ratio; CI, confidence interval. | | | | | | | | | | |
|  | | | | | | | | | | |

| **Table S4. The clinicopathologic characteristics of subgroups stratified by the nomogram** | | | | | | |
| --- | --- | --- | --- | --- | --- | --- |
| Variable |  | Patients | | | | |
|  |  | Nomogram score <135 |  | Nomogram score ≥ 135 |  | p value |
| **Primary cohort (n = 71)** |  | **n = 28** |  | **n = 43** |  |  |
| CA19-9, U/ml |  | 41(1-864) |  | 199(1-10000) |  | <0.001 |
| Tumor size, cm |  | 3.0(1.0-7.0) |  | 6.0(1.7-15.0) |  | <0.001 |
| Lymph node metastasis, yes: no |  | 1:27 |  | 27:16 |  | <0.001 |
| SDHAF2, high: medium: low |  | 1:4:23 |  | 15:24:4 |  | <0.001 |
| MRPL11, high: medium: low |  | 2:7:19 |  | 23:11:9 |  | <0.001 |
| COX8A, high: medium: low |  | 2:3:23 |  | 18:22:3 |  | <0.001 |
|  |  |  |  |  |  |  |
| **Validation cohort (n = 34)** |  | **n = 12** |  | **n = 22** |  |  |
| CA19-9, U/ml |  | 48(13-325) |  | 1335(16-10000) |  | <0.001 |
| Tumor size, cm |  | 3.0(1.2-9.3) |  | 7.2(3.5-10.0) |  | <0.001 |
| Lymph node metastasis, yes: no |  | 0:12 |  | 8:14 |  | 0.049 |
| SDHAF2, high: medium: low |  | 0:3:9 |  | 12:6:4 |  | 0.001 |
| MRPL11, high: medium: low |  | 0:3:9 |  | 8:11:3 |  | 0.001 |
| COX8A, high: medium: low |  | 0:4:8 |  | 14:8:0 |  | <0.001 |
| Abbreviations: |  |  |  |  |  |  |
| CA19-9, Carbohydrate Antigen 19-9. | | | | | | |
